# Supplementary material for: Synaptotagmin 2 Is the Fast Ca2+ Sensor at a Central Inhibitory Synapse
Source: Cell Rep. 2017 Jan 17;18(3):723–36. doi: 10.1016/j.celrep.2016.12.067 (PMC5276807; doi:10.1016/j.celrep.2016.12.067)
Supplement: Document S1. Figures S1–S7 and Tables S1 and S2 [file mmc1.pdf]

Cell Reports, Volume *18*

Supplemental Information

Synaptotagmin 2 Is the Fast Ca^{2+} Sensor at a Central Inhibitory Synapse

Chong Chen, Itaru Arai, Rachel Satterfield, Samuel M. Young, Jr., and Peter Jonas

Figure S1. Syt1 expression in cerebellar synapses of Syt2$^{+/+}$ and Syt2$^{-/-}$ mice, related to Figure 1.

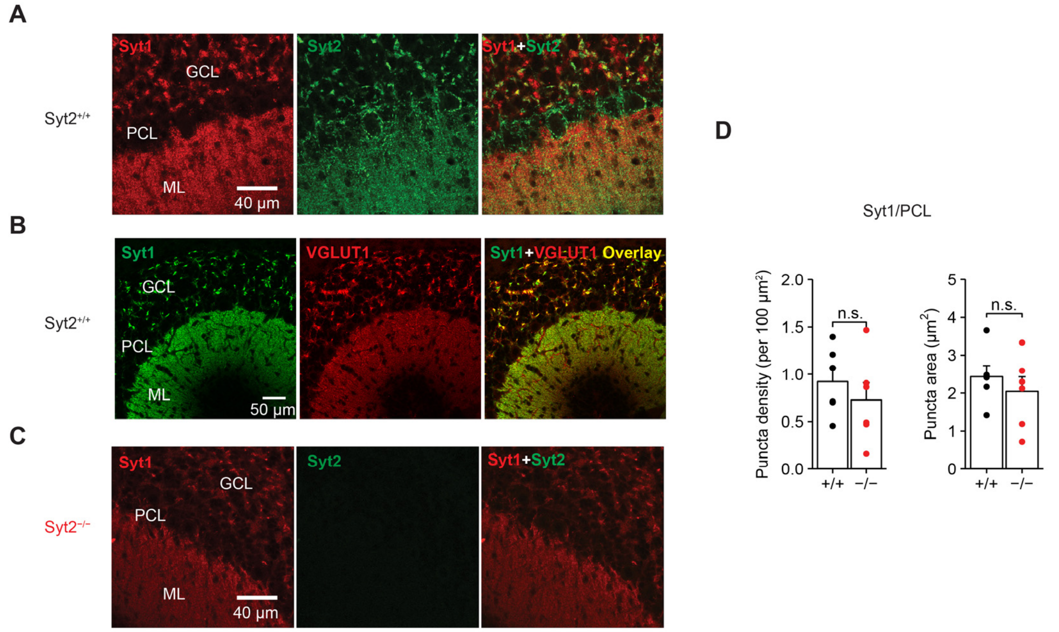

(A) Light-micrographs of cerebellar cortex, showing immunolabeling for Syt1 (left), Syt2 (center), and overlay (right).

(B) Light-micrographs of cerebellar cortex, showing immunolabeling for Syt1 (left), VGLUT1 (center), and overlay (right). Both (A) and (B) were obtained from Syt2$^{+/+}$ mice; single confocal sections.

(C) Similar analysis as in (A), but for Syt2$^{-/-}$ mouse.

(D) Summary bar graphs of puncta density (left) and puncta cross-sectional area (right) in the Purkinje cell layer (PCL). Bars represent mean ± SEM, circles indicate data from individual experiments. Note that both puncta density and area are very similar in Syt2$^{+/+}$ and Syt2$^{-/-}$ mice, suggesting lack of compensatory upregulation.

Figure S2. Similar infection and presynaptic protein levels for HdAd-Syt2 and HdAd-Syt1, related to Figure 5 and 6.

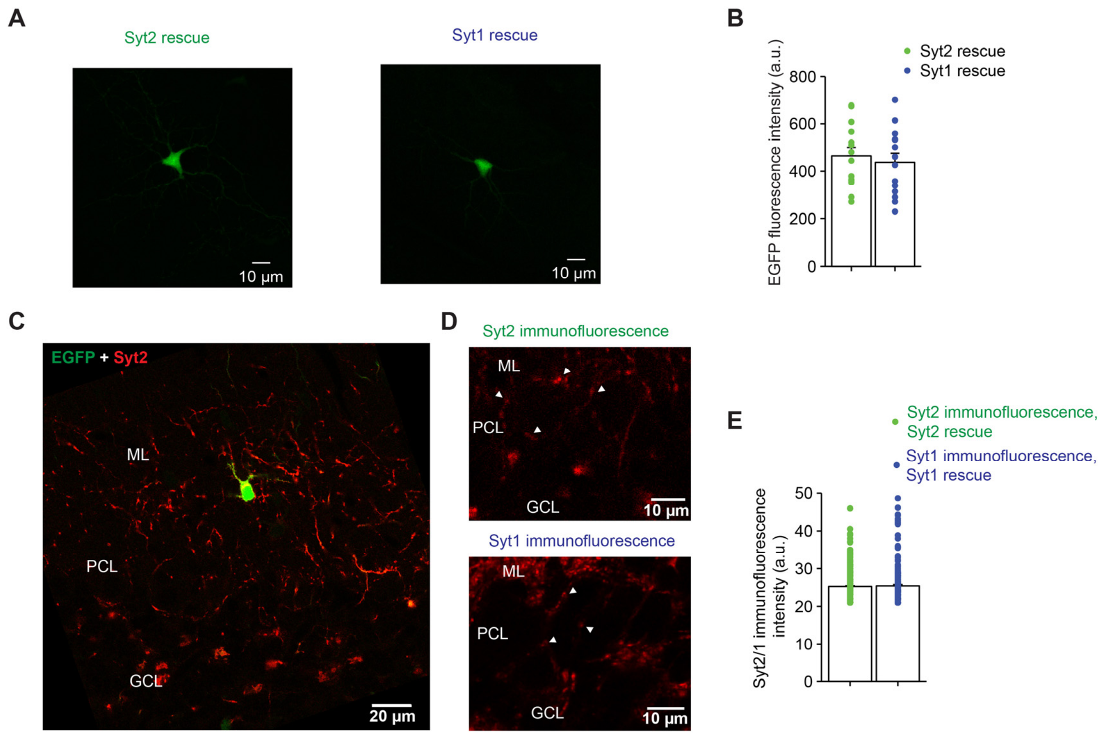

(A) Light-micrographs of cerebellar cortex, showing EGFP fluorescence in a BC in a Syt2$^{-/-}$ mouse after HdAd-Syt2 (left) or HdAd-Syt1 rescue (right).

(B) EGFP fluorescence intensity of cell bodies of HdAd-infected putative BCs in the inner molecular layer. Note that EGFP fluorescence intensity was similar (P = 0.63; 14 and 14 cells, respectively; confocal imaging performed with identical settings).

(C) Overview light micrograph of cerebellar cortex in a Syt2$^{-/-}$ mouse after infection with HdAd-Syt2. Green, EGFP fluorescence; red, Syt2 immunoreactivity. Single confocal section.

(D) High resolution light micrographs of Syt2 immunoreactivity after Syt2 rescue (top) and Syt1 immunoreactivity after Syt1 rescue (bottom) in the Purkinje cell layer. Note that both synaptotagmins are expressed after viral rescue, whereas synaptotagmin immunoreactivity is absent in Syt2$^{-/-}$ animals without rescue (Figure S1C).

(E) Quantitative analysis of Syt2 immunofluorescence after Syt2 rescue (green) and Syt1 immunofluorescence after Syt1 rescue (blue) in Syt2$^{-/-}$ animals. Measurements were made in presynaptic terminals on Purkinje cell somata, representing putative BC terminals. Fluorescence intensity was determined in the same slices following double injections into the same animal under similar conditions (identical laser illumination,

identical detector gain, similar distance from slice surface). Note that the fluorescence intensities were similar for Syt2 and Syt1 ($P = 0.58$).

Figure S3. Rescue of functional properties of synaptic transmission with HdAd-Syt2 and HdAd-Syt1, related to Figure 5 and 6.

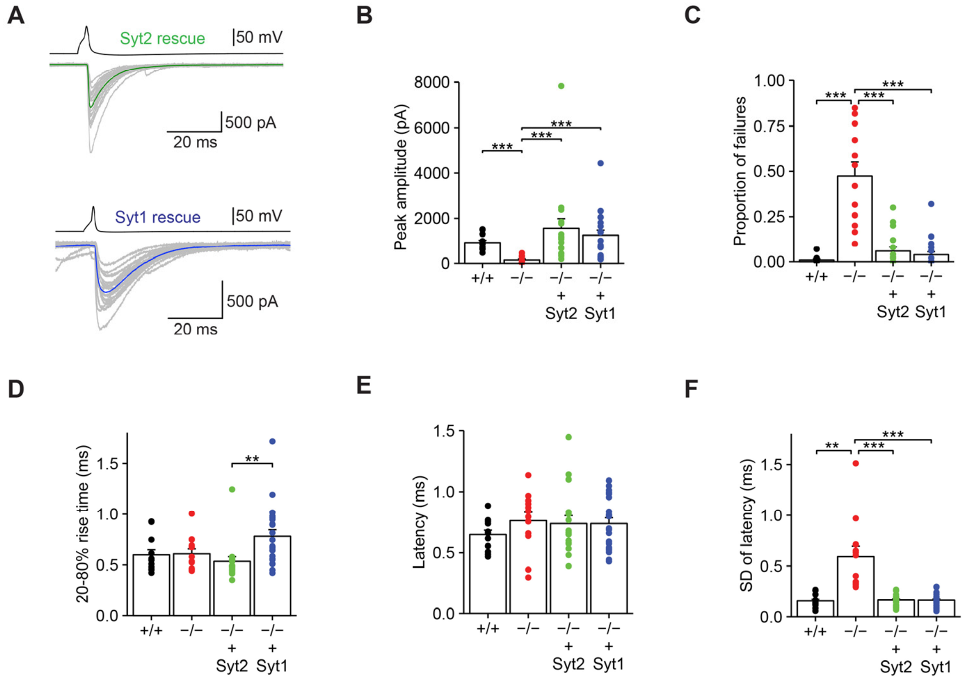

(A) Original traces of unitary IPSCs after infection of the presynaptic neuron with HdAd-Syt2 (left) or HdAd-Syt1 (right). Upper traces represent presynaptic AP, lower traces show 20 consecutive IPSC traces (gray), superimposed with the average (green and blue, respectively). Note that HdAd-Syt2 and HdAd-Syt1 were equally effective in rescuing the peak amplitude of evoked IPSCs.

(B–F) Comparison of IPSC peak amplitude (B), proportion of failures (C), 20–80% rise time (D), latency (E), and standard deviation of latency (F). Black, data from Syt2$^{+/+}$ synapses; red, data from Syt2$^{-/-}$ synapses; green, HdAd-Syt2 rescue; blue, HdAd-Syt1 rescue (both on Syt2$^{-/-}$ background). Bars represent mean ± SEM, circles indicate data from individual experiments. Note that both HdAd-Syt2 and HdAd-Syt1 similarly rescued peak amplitude, proportion of failures, latency, and standard deviation of latency, but differentially rescued the 20–80 % rise time.

Figure S4. Determining quantal size at BC–PC synapses, related to Figure 6.

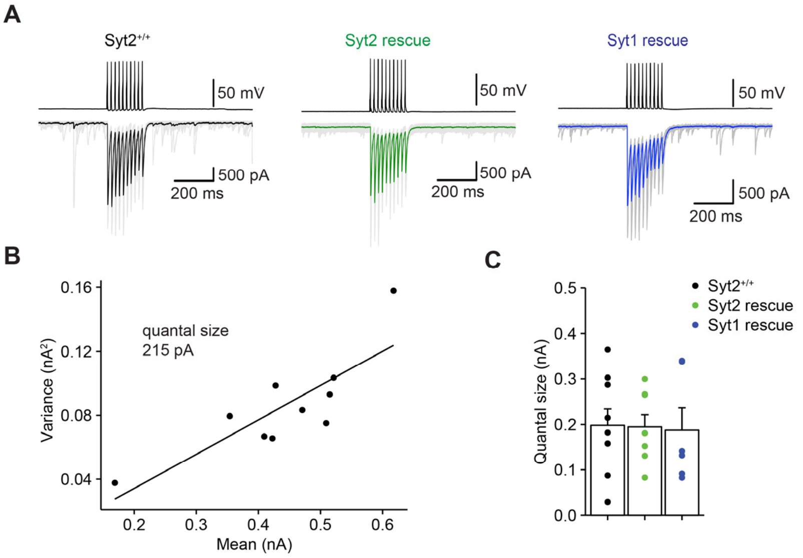

(A) Evoked IPSCs following a 50-Hz train of ten stimuli given to the presynaptic BC. Left, Syt2$^{+/+}$; center, Syt2 rescue; right, Syt1 rescue (both in Syt2$^{-/-}$ mice).

(B) Plot of variance against mean amplitude for each IPSC evoked by train stimulation. Data from a representative experiment (from a Syt2$^{+/+}$ mouse). Data points were fit by linear regression. The line slope, corresponding to quantal size, was 215 pA. Linearity of the data is consistent with constant quantal size during train stimulation.

(C) Summary bar graph of quantal size for Syt2$^{+/+}$, HdAd-Syt2 rescue, and HdAd-Syt1 rescue (both in Syt2$^{-/-}$ mice). Data from 9, 8, and 6 pairs.

Figure S5. Estimates of pool size and refilling rates are unlikely to be distorted by postsynaptic receptor saturation or desensitization, related to Figure 6.

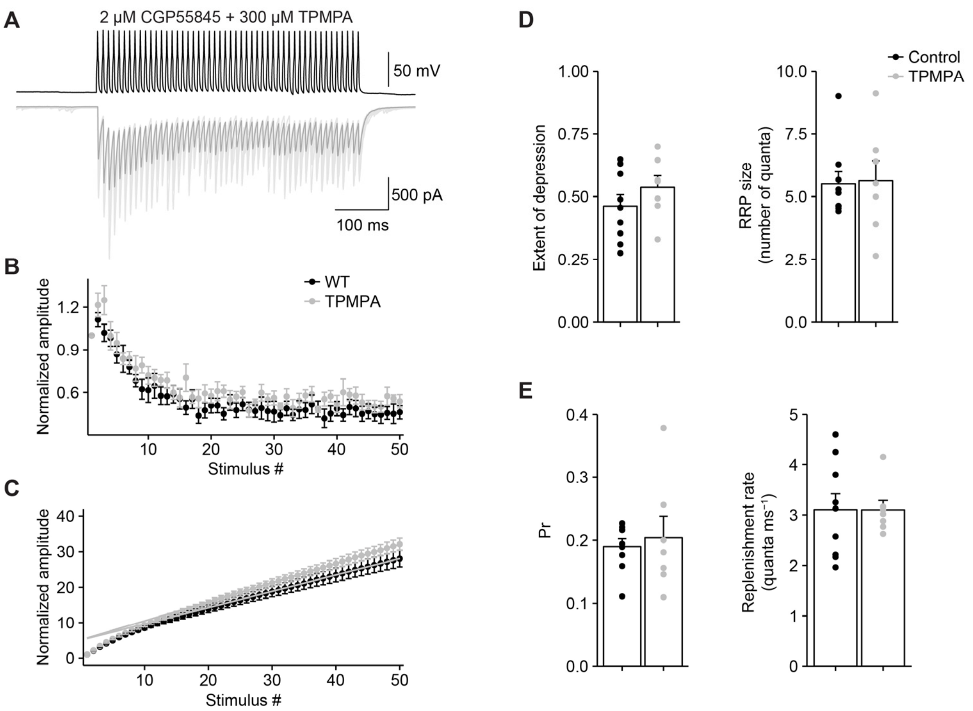

(A) Presynaptic APs (top) and evoked IPSCs (bottom) in the presence of 300 μM of low-affinity $GABA_A$ receptor antagonist TPMPA. 2 μM CGP55845 was further added to minimize effects of TPMPA on transmitter release via presynaptic $GABA_B$ receptors. 10 consecutive individual traces (light gray) and the corresponding average trace (dark gray) are shown superimposed.

(B, C) Plot of normalized IPSC peak amplitude against stimulus number (B, non-cumulative amplitude; C, cumulative plot). Black, data in control; gray, in the presence of 300 μM TPMPA. Lines represent the results of linear regression of last ten data points. Note that the pool size, as indicated by the intersection of the regression line with the ordinate, is almost identical in the absence and presence of TPMPA.

(D, E) Summary bar graphs of the extent of depression after 50 APs (D, left), size of the readily releasable pool (RRP; D, right), release probability (P_r; E, left), and replenishment rate (E, right). Bars indicate mean ± SEM; solid circles represent data from individual experiments. Data from 9 and 7 pairs, respectively. Note that the estimates were similar for control and TPMPA (P = 0.3, 0.76, 0.76, and > 0.99). All data were obtained from wild-type synapses.

Figure S6. Reversal potential of monosynaptic EPSCs and disynaptic IPSCs evoked by parallel fiber stimulation, related to Figure 7.

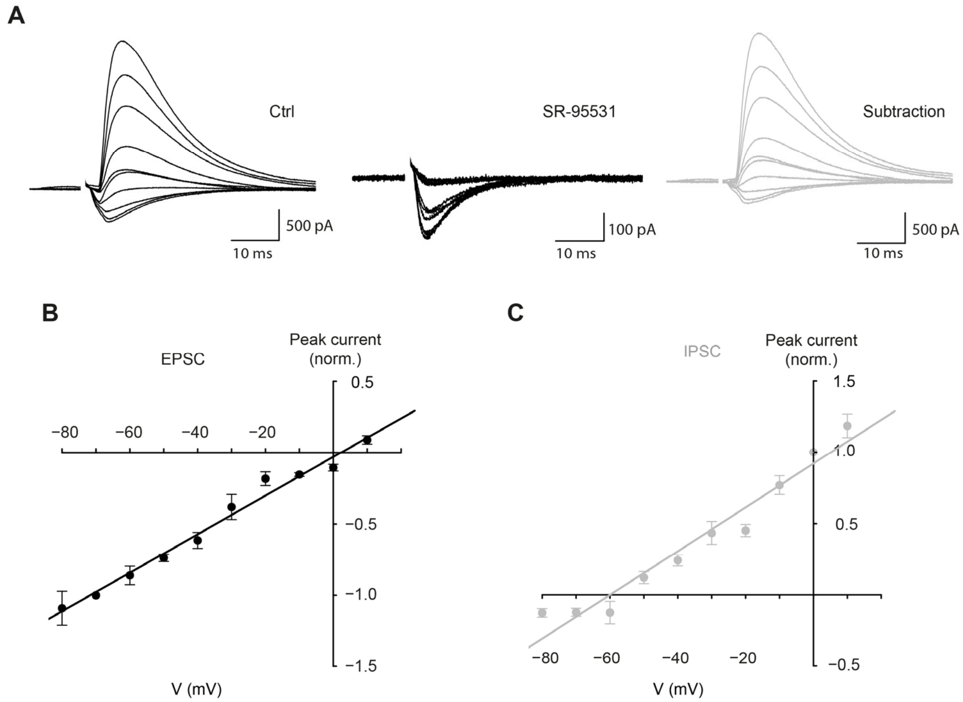

(A) Left, recording of mixed EPSCs and IPSCs; center, recording of EPSCs in the presence of 10 µM SR-95531; right, traces obtained by digital subtraction; traces were recorded at membrane potentials from −80 mV to +10 mV in 10-mV steps in a PC.

(B) Current-voltage relation for pharmacologically isolated EPSCs.

(C) Current-voltage relation for IPSCs isolated by digital subtraction. Data points indicate mean values from 4 experiments. Current amplitudes were normalized to the values at −70 mV and 0 mV, respectively. Data points were fit by linear regression, giving reversal potentials of 3.1 mV (B) and −60.0 mV (C).

Figure S7. Mechanisms of fast signaling at cerebellar BC–PC synapses, related to Figure 1–7.

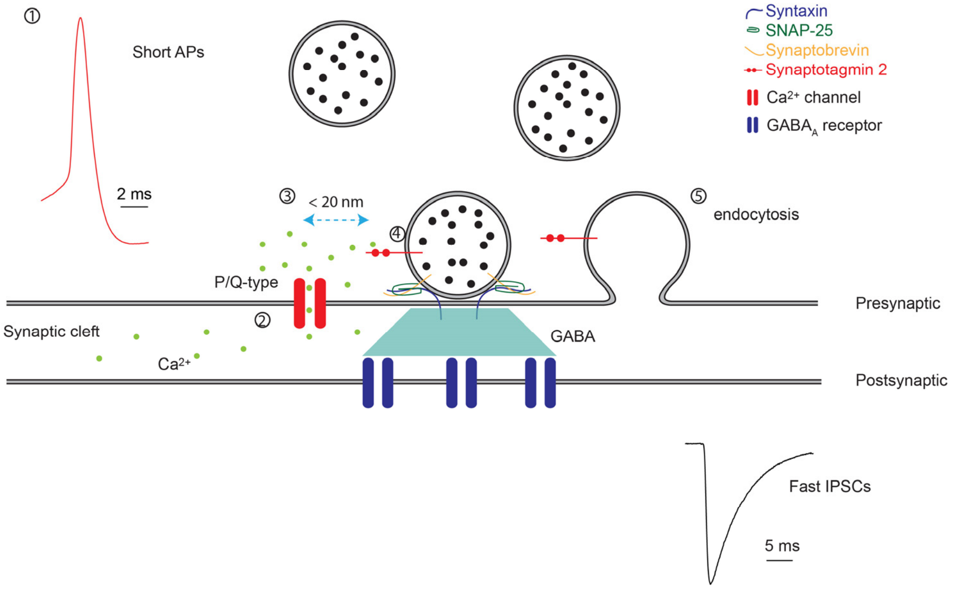

Upper left, AP recorded in a presynaptic BC. Lower right, IPSCs recorded in a postsynaptic PC. Mechanisms of rapid signaling include: (1) Short duration of presynaptic AP, (2) fast gating of presynaptic P/Q-type Ca^{2+} channels (Arai and Jonas, 2014; Li et al., 2007), (3) short coupling distance between presynaptic Ca^{2+} channels and release sensor (synaptotagmin 2, red) (Arai and Jonas, 2014), (4) fast kinetics of Syt2-mediated exocytosis (present paper), and (5) fast replenishment of releasable vesicle pool due to Syt2-mediated endocytosis (present paper).

Table S1. Functional properties of synaptic transmission at cerebellar BC–PC synapses in Syt2$^{+/+}$ and Syt2$^{−/−}$ mice, related to Figure 2, 3, and 4.

Parameter	Syt2$^{+/+}$	Syt2$^{−/−}$	P
Latency	0.65± 0.04 ms (12)	0.75 ± 0.07 ms (12)	0.1135
SD Latency	0.15± 0.02 ms (12)	0.59 ± 0.10 ms (12)	< 0.0001
20–80% rise time	0.60 ± 0.05 ms (12)	0.64 ± 0.05 ms (12)	0.5137
IPSC peak amplitude	925.1 ± 99.2 pA (12)	151.9 ± 44.2 pA (12)	< 0.0001
IPSC decay time constant	9.19 ± 0.51 ms (12)	9.53 ± 1.08 ms (12)	0.6707
Proportion of failures	0.91 ± 0.58% (12)	47.4 ± 7.7% (12)	< 0.0001
Paired-pulse ratio $IPSC_2$ / $IPSC_1$	1.06 ± 0.05 (11)	1.79 ± 0.16 (16)	0.0003
$IPSC_{10}$ / $IPSC_1$	0.61 ± 0.02 (11)	2.79 ± 0.46 (16)	< 0.0001
Miniature IPSC frequency	3.75 ± 0.42 Hz (12)	9.64 ± 1.74 Hz (13)	0.0008
Miniature IPSC peak amplitude	110.0 ±11.0 pA (12)	141.7±12.0 pA (13)	0.0868

Mean ± SEM (n, number of experiments).

Table S2. Differential helper-dependent adenovirus-mediated rescue by Syt2 and Syt1 in Syt2$^{-/-}$ mice, related to Figure 5 and 6.

Parameter	HdAd-Syt2	HdAd-Syt1	P
Latency	0.74 ± 0.06 ms (17)	0.74 ± 0.05 ms (21)	>0.9999
SD latency	0.16 ± 0.01 ms (17)	0.16 ± 0.01 ms (21)	>0.9999
20–80% rise time	0.534 ± 0.05 ms (17)	0.781 ± 0.065 ms (21)	0.0026
IPSC peak amplitude	1559.3 ± 426.4 pA (17)	1250.3 ± 225.2 pA (21)	0.9740
IPSC decay time constant	7.71 ± 0.41 ms (17)	9.27 ± 0.55 ms (21)	0.2700
Proportion of failures	5.91 ± 2.28% (17)	3.81 ± 1.67% (21)	>0.9999
Paired-pulse ratio $IPSC_2$ / $IPSC_1$	1.01 ± 0.04 (10)	0.82 ± 0.05 (10)	0.0294
$IPSC_{10}$ / $IPSC_1$	0.67 ± 0.08 (10)	0.39 ± 0.032 (10)	0.0030
Recovery from depression τ	2.17 ± 0.31 s (13)	4.89 ± 0.79 s (12)	0.011

All experiments were performed in Syt2$^{-/-}$ mice.
Mean ± SEM (n, number of experiments).

Data included in this paper are based on recordings from a total of 137 BC–PC paired recordings (32 for experiments on Syt2$^{+/+}$, 28 for Syt2$^{-/-}$, 37 for HdAd-Syt2 rescue, and 40 for HdAd-Syt1 rescue) and 48 PC recordings (25 for mIPSC and 23 for disynaptic inhibition experiments).
