# Supplementary material for: Synaptotagmin 2 Is the Fast Ca2+ Sensor at a Central Inhibitory Synapse
Source: Cell Rep. 2017 Jan 17;18(3):723–36. doi: 10.1016/j.celrep.2016.12.067 (PMC5276807; doi:10.1016/j.celrep.2016.12.067)
Supplement: Document S2. Article plus Supplemental Information [file mmc2.pdf]

Cell Reports

Article

Synaptotagmin 2 Is the Fast Ca^{2+} Sensor at a Central Inhibitory Synapse

Graphical Abstract

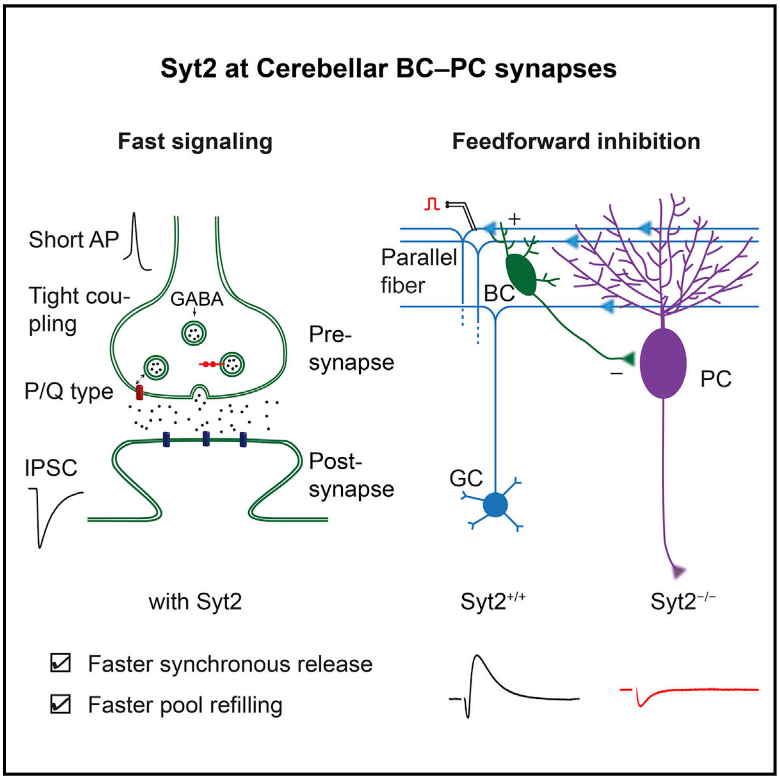

Highlights

- Syt2 is the Ca^{2+} sensor of fast transmitter release at a cerebellar GABAergic synapse
- Syt2 triggers transmitter release with faster time course than Syt1
- Syt2 ensures faster replenishment of the readily releasable pool than Syt1
- Syt2 is essential for fast feedforward inhibition in cerebellar microcircuits

Authors

Chong Chen, Itaru Arai,
Rachel Satterfield, Samuel M. Young, Jr.,
Peter Jonas

Correspondence

peter.jonas@ist.ac.at

In Brief

Chen et al. identify synaptotagmin 2 as the major Ca^{2+} sensor of transmitter release at cerebellar inhibitory synapses. Furthermore, they demonstrate that synaptotagmin 2 triggers faster release and ensures faster refilling of the vesicular pool than synaptotagmin 1.

Chen et al., 2017, Cell Reports *18*, 723–736
January 17, 2017 © 2017 The Author(s).
http://dx.doi.org/10.1016/j.celrep.2016.12.067

Cell Reports
Article

OPEN ACCESS
CellPress

Synaptotagmin 2 Is the Fast Ca^{2+} Sensor at a Central Inhibitory Synapse

Chong Chen,[1,3] Itaru Arai,[1,3,4] Rachel Satterfield,[2] Samuel M. Young, Jr.,[2] and Peter Jonas[1,5,*]
[1]IST Austria (Institute of Science and Technology Austria), Am Campus 1, 3400 Klosterneuburg, Austria
[2]Max Planck Florida Institute for Neuroscience, Research Group Molecular Mechanisms of Synaptic Function, Jupiter, FL 33458, USA
[3]Co-first author
[4]Present address: Department of Neurophysiology, Keio University School of Medicine, 35 Shinanomachi, Shinjuku-ku, Tokyo 160-8582, Japan
[5]Lead Contact
*Correspondence: peter.jonas@ist.ac.at
http://dx.doi.org/10.1016/j.celrep.2016.12.067

SUMMARY

GABAergic synapses in brain circuits generate inhibitory output signals with submillisecond latency and temporal precision. Whether the molecular identity of the release sensor contributes to these signaling properties remains unclear. Here, we examined the Ca^{2+} sensor of exocytosis at GABAergic basket cell (BC) to Purkinje cell (PC) synapses in cerebellum. Immunolabeling suggested that BC terminals selectively expressed synaptotagmin 2 (Syt2), whereas synaptotagmin 1 (Syt1) was enriched in excitatory terminals. Genetic elimination of Syt2 reduced action potential-evoked release to ~10%, identifying Syt2 as the major Ca^{2+} sensor at BC-PC synapses. Differential adenovirus-mediated rescue revealed that Syt2 triggered release with shorter latency and higher temporal precision and mediated faster vesicle pool replenishment than Syt1. Furthermore, deletion of Syt2 severely reduced and delayed disynaptic inhibition following parallel fiber stimulation. Thus, the selective use of Syt2 as release sensor at BC-PC synapses ensures fast and efficient feedforward inhibition in cerebellar microcircuits.

INTRODUCTION

γ-aminobutyric acid (GABA)-ergic interneurons play a key role in the control of activity in neuronal microcircuits. These neurons mediate feedback and feedforward inhibition and are involved in the generation of high-frequency network oscillations (Hu et al., 2014). A hallmark functional property of GABAergic interneurons, especially of parvalbumin-expressing subtypes, is the speed of signaling at their output synapses across species and brain regions (Kraushaar and Jonas 2000; Hefft and Jonas, 2005; Caillard et al., 2000; Sakaba, 2008; Arai and Jonas, 2014). Several molecular and subcellular factors may underlie submillisecond signaling at GABAergic synapses. The selective use of P/Q-type Ca^{2+} channels in presynaptic terminals will contribute, because these channels activate and deactivate very rapidly (Li et al., 2007; Hefft and Jonas, 2005; Arai and Jonas, 2014). The tight coupling between Ca^{2+} channels and Ca^{2+} sensors of exocytosis will be relevant, because such "nanodomain coupling" will reduce diffusional delays (Bucurenciu et al., 2008; Eggermann et al., 2011; Chen et al., 2015). Finally, the activation and deactivation kinetics of the Ca^{2+} sensors of exocytosis (presumably synaptotagmins) may be important for the speed of synaptic transmission (Chapman, 2002; Südhof, 2002; Koh and Bellen, 2003; Xu et al., 2007; Kochubey et al., 2016).

However, the molecular identity of the Ca^{2+} sensor at GABAergic synapses has not been determined yet. The mammalian genome encodes 16 synaptotagmins, eight of which bind Ca^{2+} (Syt1, 2, 3, 5, 6, 7, 9, and 10; Chapman, 2002; Südhof, 2002), and three of which act as fast release sensors (Syt1, Syt2, and Syt9; Xu et al., 2007; Kochubey et al., 2016). Which of these synaptotagmins are involved in transmitter release at GABAergic synapses remains unclear. Genetic labeling and immunohistochemistry experiments suggested the expression of Syt2 in GABAergic interneurons, probably parvalbumin-expressing subtypes (Pang et al., 2006a; Fox and Sanes, 2007; Sommeijer and Levelt, 2012). However, genetic elimination of Syt1 reduced release at output synapses of fast-spiking, parvalbumin-expressing interneurons in the hippocampus to ~50% of control value (Kerr et al., 2008), suggesting that multiple synaptotagmins, possibly Syt1 and Syt2, work in concert.

How different synaptotagmin isoforms shape the time course of transmitter release is controversial. Recombinant expression of different synaptotagmin isoforms in cultured neurons suggested marked kinetic differences among Syt1, Syt2, and Syt9, with Syt2 mediating the fastest and Syt9 the slowest time course of release (Xu et al., 2007). However, expression of different synaptotagmin isoforms in the calyx of Held revealed no significant differences in release kinetics between Syt2 and Syt1 (Kochubey et al., 2016). Furthermore, Syt2 mediated slower vesicle fusion kinetics than Syt1 in chromaffin cells (Nagy et al., 2006) and showed slower kinetics in comparison to Syt1 in liposome binding-unbinding assays (Hui et al., 2005). Thus, how the differential expression of synaptotagmin isoforms (Mittelsteadt et al., 2009) relates to the speed of synaptic transmission has not been defined.

 © 2017 The Author(s).
This is an open access article under the CC BY license (http://creativecommons.org/licenses/by/4.0/).

OPEN
ACCESS
CellPress

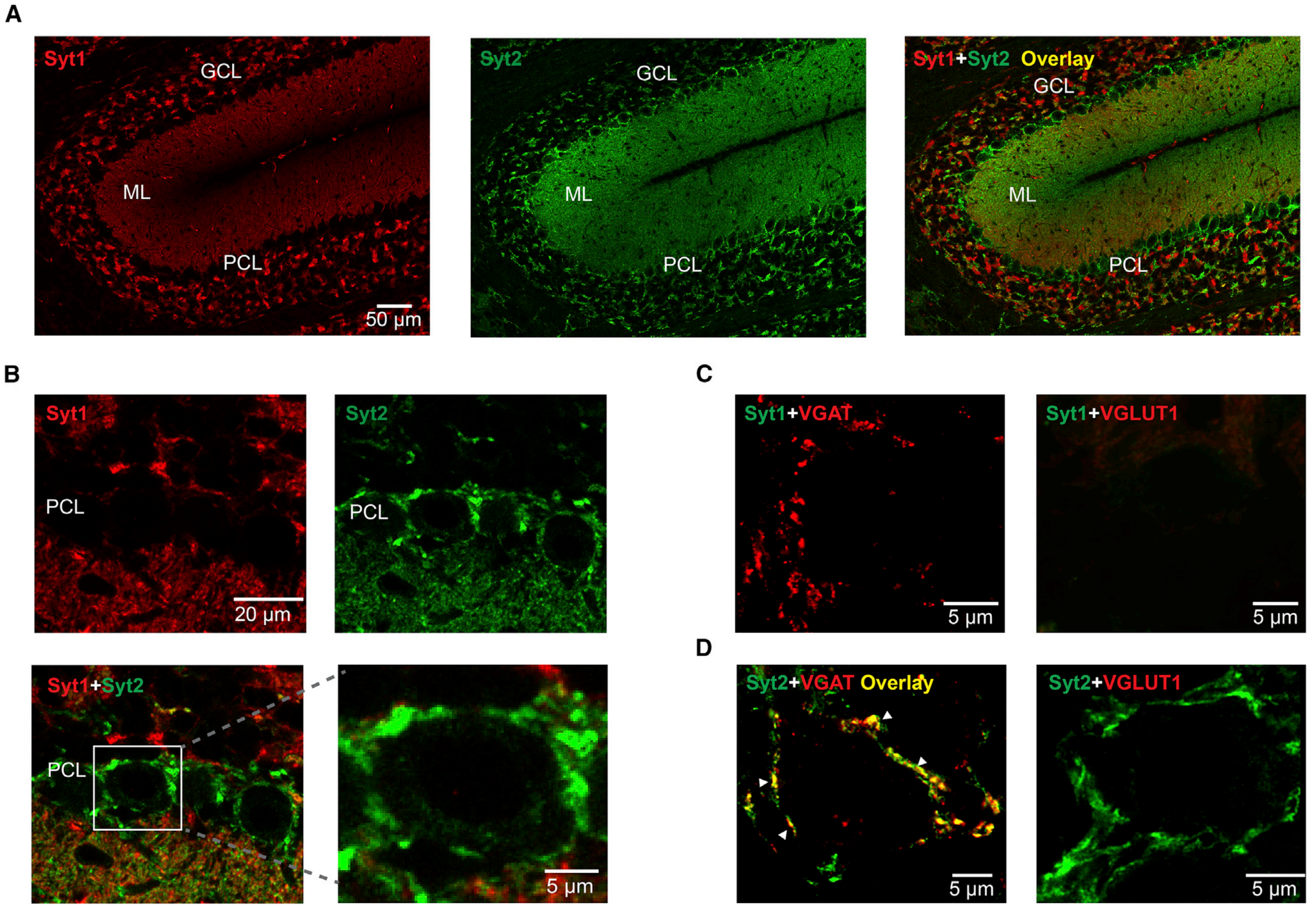

Figure 1. Differential Expression of Syt1 and Syt2 between Excitatory and Inhibitory Presynaptic Terminals in Cerebellum

(A) Light-micrographs of cerebellar cortex, showing immunolabeling for synaptotagmin 1 (Syt1, left), synaptotagmin 2 (Syt2, center), and overlay (right) from a wild-type mouse; single confocal sections.

(B) Similar to (A), but at higher magnification. Note that putative BC terminals surrounding PC somata are only immunoreactive for Syt2, but not for Syt1.

(C) Colocalization of Syt1 and VGAT (left) and VGLUT1 (right). Note the absence of Syt1 immunoreactivity in inhibitory terminals surrounding PCs.

(D) Similar colocalization analysis as shown in (C), but for Syt2. Note high Syt2 immunoreactivity in putative BC terminals (arrowheads).

ML, molecular layer; PCL, Purkinje cell layer; GCL, granule cell layer.

The cerebellar basket cell (BC)-Purkinje cell (PC) synapse seems to be an ideal synapse to address these questions. First, this synapse showed high connectivity and strictly perisomatic location (Arai and Jonas, 2014), enabling us to examine the time course of release with microsecond temporal precision. Second, in comparison to hippocampus and neocortex, interneuron diversity is more limited, making it easier to dissect the functional contribution of synaptotagmin isoforms by comparison of wild-type and knockout synapses. Third, this synapse is amenable to genetic manipulation (such as virus injection), because of the superficial location of the cerebellar microcircuits. Finally, the effects of Syt2 deletion can be tested in acute slices. This is important, because the effects of synaptotagmins may depend on the network environment (Liu et al., 2009). We discovered that genetic deletion of Syt2 largely abolished fast transmitter release, and that Syt2 generated faster release kinetics and faster pool refilling than Syt1 in rescue experiments.

RESULTS

Differential Expression of Syt1 and Syt2 between Excitatory and Inhibitory Presynaptic Terminals in Cerebellum

Previous expression analysis showed that several synaptotagmin isoforms are expressed in the cerebellum, with strongest expression of Syt1 and Syt2 (Mittelsteadt et al., 2009). To determine the molecular identity of the Ca^{2+} sensor at cerebellar BC-PC synapses, we examined synaptotagmin immunoreactivity in BC terminals, using specific antibodies against either Syt1 or Syt2 (Fox and Sanes, 2007; Kochubey et al., 2016; Figure 1). Although Syt1 immunoreactivity was abundant in both the granule cell layer and the molecular layer, putative presynaptic BC terminals surrounding PC somata were completely devoid of fluorescent labeling (Figures 1A and 1B). In contrast, putative BC terminals were strongly immunopositive for Syt2 (Figures 1A and 1B). Syt2 labeling was completely eliminated in the $Syt2^{-/-}$

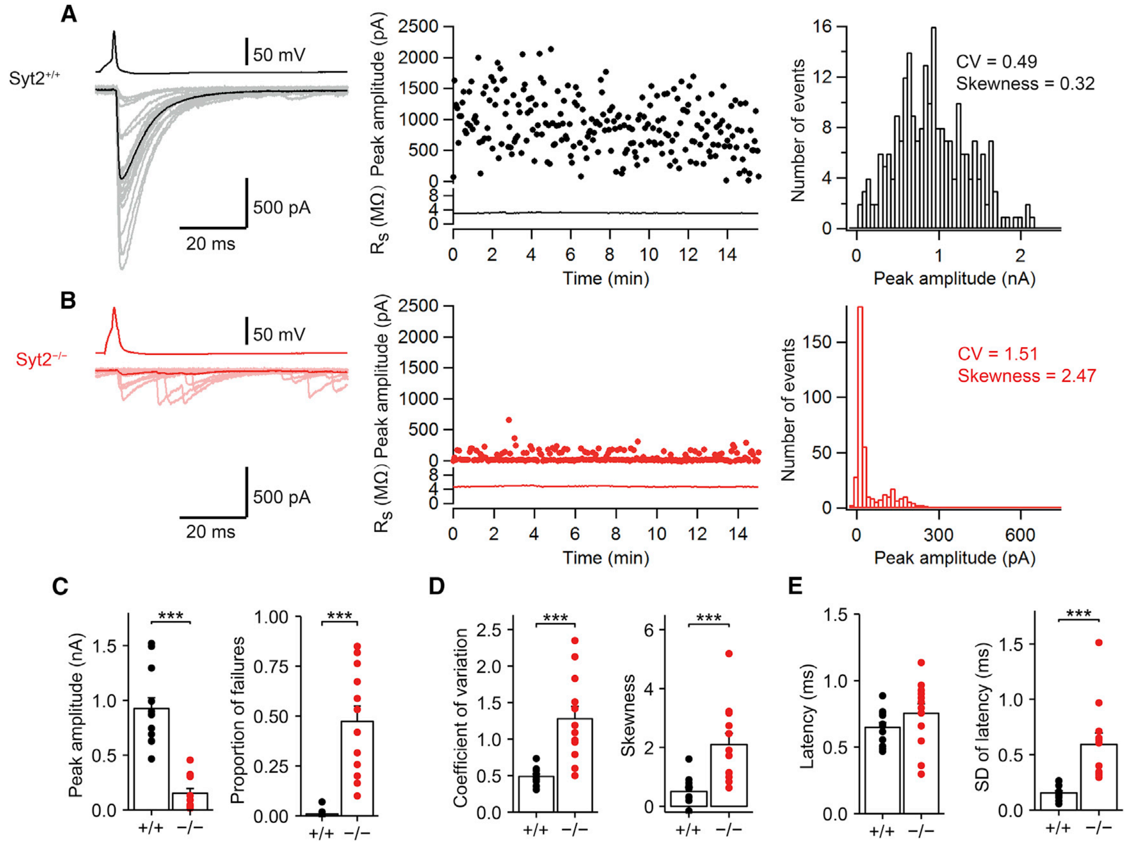

Figure 2. Genetic Elimination of Syt2 Severely Reduces Transmitter Release at the BC-PC Synapse

(A) Left, presynaptic AP (black), individual evoked IPSCs (gray), and average IPSC (black) recorded from a $Syt2^{+/+}$ synapse. Center, plot of IPSC peak amplitude and postsynaptic series resistance (R_S) against experimental time. Right, peak amplitude histogram from the same pair, obtained from 235 unitary IPSCs. The number of failures was 2 in this experiment.

(B) Similar experiment as shown in (A) but recorded from a $Syt2^{-/-}$ synapse (red, light red, and red). Histogram shown on the right was obtained from 400 unitary IPSCs. The number of failures was 279.

(C–E) Genetic elimination of Syt2 reduces release probability. Summary bar graph of peak amplitude (C, left), proportion of failures (C, right), coefficient of variation (CV, D, left), skewness (D, right), latency (E, left), and SD of latency (E, right) for $Syt2^{+/+}$ (black) and $Syt2^{-/-}$ synapses (red). Bars represent mean ± SEM; points indicate data from individual experiments. Data are from 12 pairs ($Syt2^{+/+}$) and 12 pairs ($Syt2^{-/-}$).

mice, demonstrating the specificity of the antibody (Figure S1). Double labeling with antibodies against Syt1 and Syt2 revealed that, despite the abundant expression of both isoforms in the cerebellum, colocalization of immunoreactivities was minimal, indicating expression in nonoverlapping populations of synapses (Figure 1B). Double labeling with antibodies against one of the synaptotagmins and the vesicular GABA transporter (VGAT) or vesicular glutamate transporter (VGLUT1) demonstrated that in the cerebellum Syt1 was exclusively expressed at excitatory synapses (Figure S1B; see Figure 1C), whereas Syt2 was largely confined to inhibitory synapses (Figure 1D). These results suggest that Syt2 may constitute the main Ca^{2+} sensor for fast transmitter release at inhibitory BC-PC synapses.

Syt2 Deletion Severely Reduces Action Potential-Evoked Release at BC-PC Synapses

To directly test the hypothesis that Syt2 is the Ca^{2+} sensor of exocytosis at BC-PC synapses, we studied the effects of genetic elimination. To achieve this, we compared the properties of unitary inhibitory postsynaptic currents (IPSCs) between wild-type ($Syt2^{+/+}$) and Syt2-deficient ($Syt2^{-/-}$) BC-PC synapses in 14- to 16-day-old mice (Figure 2; Table S1). Genetic elimination of Syt2 reduced the peak amplitude of evoked IPSCs at BC-PC synapses to 16.4% of the wild-type control value (925.1 ± 99.2 pA in $Syt2^{+/+}$ synapses; 151.9 ± 44.2 pA in $Syt2^{-/-}$ synapses, 12 pairs in both cases; $p < 0.0001$; Figures 2A–2C). In parallel, genetic elimination of Syt2 markedly increased the proportion of failures, from 0.9% ± 0.6% in $Syt2^{+/+}$ mice to 47.4% ± 7.7% in $Syt2^{-/-}$ mice ($p < 0.0001$; Figure 2C), confirming a presynaptic change. These results identify Syt2 as the main functional Ca^{2+} sensor for synchronous transmitter release at inhibitory BC-PC synapses.

To reveal the mechanisms by which genetic elimination of Syt2 reduces the amplitude of evoked IPSCs, we further examined the coefficient of variation (CV) and the skewness of IPSC peak amplitudes. Syt2 deletion increased the CV, from 0.49 ± 0.03 to 1.28 ± 0.17 (12 pairs in both cases; $p < 0.0001$; Figure 2D). Furthermore, genetic elimination of Syt2 increased the

OPEN ACCESS
CellPress

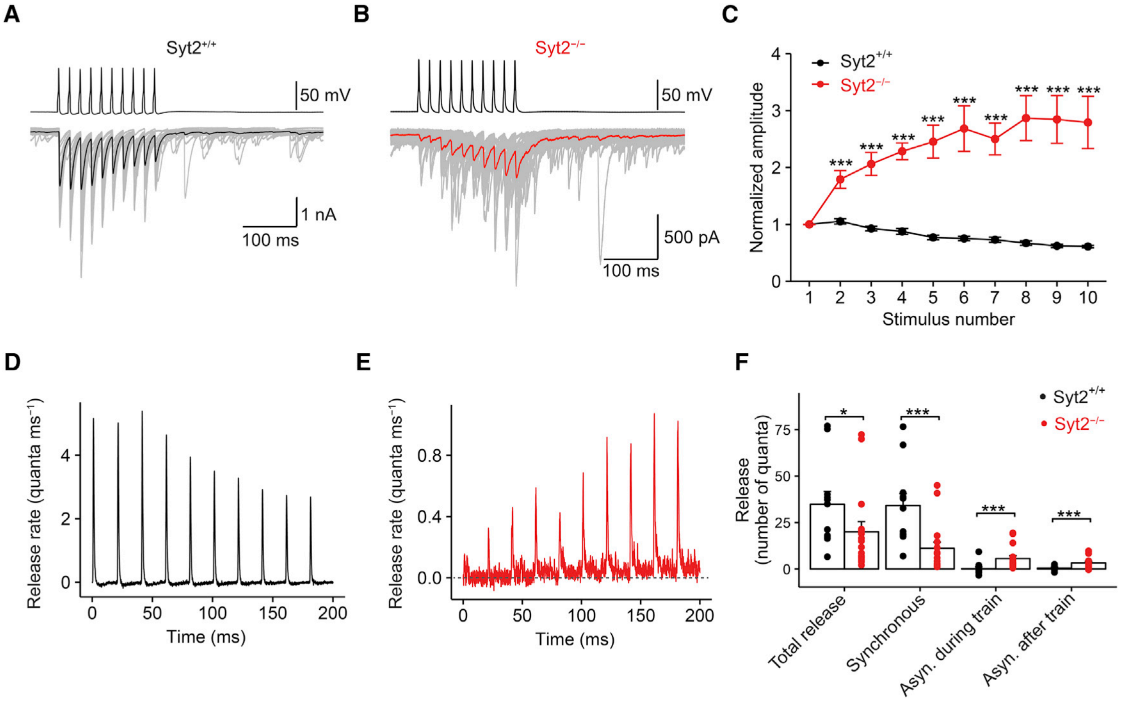

Figure 3. Genetic Elimination of Syt2 Selectively Abolishes Synchronous Release

(A and B) Trains of ten presynaptic APs at 50 Hz and corresponding IPSCs recorded from a Syt2$^{+/+}$ synapse (A) and a Syt2$^{-/-}$ synapse (B). 20 consecutive IPSC traces (gray) and the average trace (black and red, respectively) are shown superimposed.

(C) Plot of normalized IPSC amplitude ($IPSC_n$ / $IPSC_1$) against IPSC number (n) for Syt2$^{+/+}$ (black circles) and Syt2$^{-/-}$ synapses (red circles). Error bars indicate SEM.

(D and E) Corresponding release rate during the AP train obtained by deconvolution for a Syt2$^{+/+}$ synapse (D) and a Syt2$^{-/-}$ synapse (E; horizontal dashed line indicates a release rate of 0).

(F) Summary bar graph of cumulative release (total release, synchronous release, asynchronous release during the train, and asynchronous release after the train). Bars represent mean ± SEM; points indicate data from individual experiments.

All data were obtained with trains of ten APs, applied at a frequency of 50 Hz. Data in (C) and (F) are from 11 pairs (Syt2$^{+/+}$) and 16 pairs (Syt2$^{-/-}$).

skewness, from 0.50 ± 0.14 in Syt2$^{+/+}$ mice to 2.10 ± 0.38 in Syt2$^{-/-}$ mice ($p < 0.0001$; Figure 2D). Taken together, these results suggest that Syt2 deletion reduces release probability without changing the number of release sites (Kerr et al., 2008). Finally, we quantified the latency and its SD, a measure of temporal precision of transmitter release. Whereas latency was not significantly different (0.65 ± 0.04 ms in Syt2$^{+/+}$; 0.75 ± 0.07 ms in Syt2$^{-/-}$; $p = 0.11$; Figure 2E), SD of latency markedly increased, from 0.15 ± 0.02 ms to 0.59 ± 0.10 ms ($p < 0.0001$; Figure 2E). Thus, the residual component in Syt2$^{-/-}$ mice appeared more asynchronous. Compensatory increases in the expression of Syt1 were not detected in Syt2$^{-/-}$ mice (Figures S1C and S1D), suggesting that the residual component was mediated by a different Ca^{2+} sensor, e.g., Syt7 (Bacaj et al., 2013; Jackman et al., 2016). In conclusion, deletion of the Syt2 gene resulted in a massive reduction of synchronous transmitter release at cerebellar BC-PC synapses, caused by a reduction in release probability.

Effects of Syt2 Deletion on Short-Term Dynamics and Asynchronous Release

Next, we studied the effects of genetic elimination of Syt2 on IPSCs evoked by trains of presynaptic stimuli (Figure 3). In Syt2$^{+/+}$ synapses, trains of ten presynaptic action potentials (APs) at 50 Hz induced a significant depression of IPSC peak amplitude and a minimal amount of asynchronous release during and after the stimulus train (Figures 3A and 3C), consistent with previous observations (Caillard et al., 2000; Sakaba, 2008; Arai and Jonas, 2014). In contrast, in Syt2$^{-/-}$ synapses, IPSC peak amplitudes showed a marked facilitation (Figures 3B and 3C), and the frequency of asynchronous release was increased (Figure 3B). To quantify synchronous and asynchronous release components, we analyzed release during train stimulation by deconvolution (Figures 3D and 3E). Synchronous release was quantified in the time interval of 5 ms following the AP, whereas asynchronous release was measured in a time window 15–20 ms following each presynaptic stimulus. Syt2 deletion reduced the cumulative release of the synchronous component from 34.1 ± 6.5 quanta in Syt2$^{+/+}$ mice to 11.1 ± 3.3 quanta in Syt2$^{-/-}$ mice (11 and 16 pairs, respectively; $p = 0.0009$; Figure 3F). In contrast, Syt2 deletion increased asynchronous release during the train from 0.14 ± 0.99 quanta in Syt2$^{+/+}$ mice to 5.62 ± 1.53 quanta in Syt2$^{-/-}$ mice (11 and 16 pairs, respectively; $p = 0.0001$; Figure 3F). Similarly, genetic elimination of Syt2 increased asynchronous release after the train from 0.52 ± 0.38 quanta in Syt2$^{+/+}$ mice to 3.31 ± 0.81 quanta in Syt2$^{-/-}$ mice (11 and 16

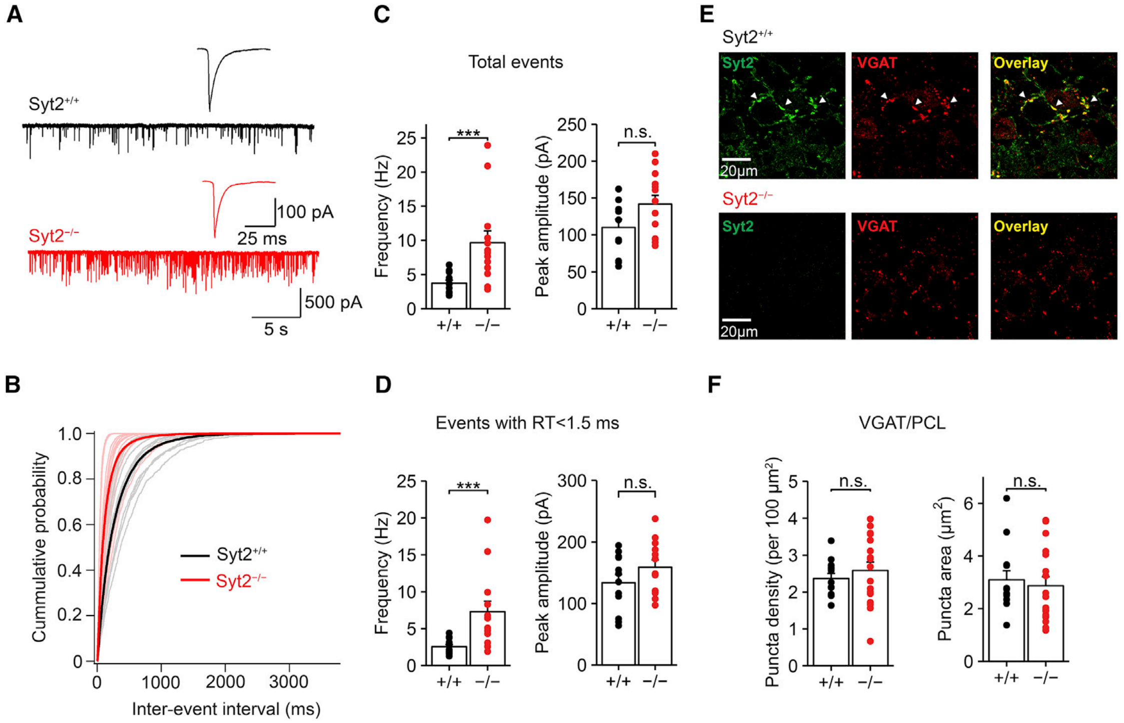

Figure 4. Genetic Elimination of Syt2 Increases mIPSC Frequency

(A) Traces of mIPSCs in a PC at −70 mV in Syt2$^{+/+}$ slices (top, black traces) and Syt2$^{-/-}$ slices (bottom, red traces). 1 μM TTX, 20 μM D-AP5, and 10 μM CNQX were added to external solution to block spontaneous AP firing and excitatory synaptic activity. Inset shows average mIPSC at expanded timescale (averages from 1,073 and 2,132 single events).

(B) Cumulative histograms of mIPSC inter-event interval (gray: from individual PCs of Syt2$^{+/+}$ mice; black: average of Syt2$^{+/+}$ data; light red: from individual PCs of Syt2$^{-/-}$ mice; red: average of Syt2$^{-/-}$ data).

(C) Comparison of mIPSC frequency (left) and peak amplitude (right) between Syt2$^{+/+}$ and Syt2$^{-/-}$ mice. Bars represent mean ± SEM; points indicate data from individual experiments. Data were obtained from 12 PCs (Syt2$^{+/+}$) and 13 PCs (Syt2$^{-/-}$).

(D) Similar data as shown in (C), but for mIPSCs with 20%–80% rise time <1.5 ms, presumably corresponding to synaptic events generated by BC terminals (Roth and Häusser, 2001).

(E) Confocal light-micrograph of Syt2 immunoreactivity (left), VGAT immunoreactivity (center), and overlay (right) in Syt2$^{+/+}$ (top) and Syt2$^{-/-}$ (bottom) mice; single confocal sections.

(F) Summary bar graphs of puncta density (left) and puncta cross-sectional area (right) in the PC layer. Bars represent mean ± SEM; circles indicate data from individual experiments.

pairs, respectively; $p = 0.0033$; Figure 3F). These results demonstrate that Syt2 selectively mediates synchronous release, whereas asynchronous release appears to be mediated by a different sensor, e.g., Syt7 (Bacaj et al., 2013; Jackman et al., 2016).

Elevated Frequency of Spontaneous Release in Syt2$^{-/-}$ Mice

Previous studies showed that deletion of synaptotagmins increases the frequency of spontaneous release, suggesting a clamping function of synaptotagmin (Littleton et al., 1994; Pang et al., 2006b; Chicka et al., 2008; Giraudo et al., 2006; Kerr et al., 2008). However, these experiments have mostly been performed in culture conditions, in which compensatory changes in connectivity or homeostatic mechanisms may occur (Liu et al., 2014). To test the clamping hypothesis in the intact circuit, we compared the frequency of miniature IPSCs (mIPSCs) in PCs between Syt2$^{+/+}$ and Syt2$^{-/-}$ mice (Figure 4). mIPSCs were recorded in pharmacological isolation in the presence of Na^{+} channel and glutamate receptor blockers (1 μM tetrodotoxin [TTX]; 10 μM 6-cyano-7-nitroquinoxaline-2,3-dione, CNQX; 20 μM D-2-amino-5-phosphonopentanoic acid, D-AP5) and were detected with a template matching algorithm (Pernía-Andrade et al., 2012; Figure 4A). Genetic elimination of Syt2 resulted in an ~2.5-fold increase in the frequency of mIPSCs in PCs, from 3.75 ± 0.42 Hz in Syt2$^{+/+}$ mice (12 cells) to 9.64 ± 1.74 Hz in Syt2$^{-/-}$ mice (13 cells; $p = 0.0008$; Figures 4B and 4C). In contrast, the amplitude of mIPSCs was unchanged (110.0 ± 11.0 pA in Syt2$^{+/+}$ versus 141.7 ± 12.0 pA in Syt2$^{-/-}$; $p = 0.087$; Figure 4C). These results are consistent with a clamping function of Syt2 at GABAergic synapses.

To distinguish mIPSCs generated at BC synapses from those at stellate cell synapses, we analyzed the 20%–80% rise time of mIPSCs, an indicator of synaptic location. When the analysis was restricted to IPSCs with a 20%–80% rise time of <1.5 ms, likely to be generated by synapses in the inner third of the

molecular layer (Roth and Häusser, 2001), Syt2 deletion resulted in an ~2.5-fold increase in the frequency of mIPSCs, from 2.54 ± 0.30 Hz to 7.28 ± 1.43 Hz (12 and 13 cells, respectively; $p = 0.0005$; Figure 4D). Furthermore, the amplitude of mIPSCs was unchanged ($p = 0.27$; Figure 4D). To rule out that changes in inhibitory connectivity confounded these observations, we labeled GABAergic synaptic sites with antibodies against VGAT and quantified the density of immunopositive puncta in Syt2$^{+/+}$ and Syt2$^{-/-}$ mice in the PC layer (Figures 4E and 4F). On average, the number of VGAT-positive puncta per 100 μm^2 in single confocal sections was 2.37 in Syt2$^{+/+}$ and 2.59 in Syt2$^{-/-}$ mice (Figure 4F, left; $p = 0.39$). Likewise, the puncta area was not significantly different (Figure 4F, right; $p = 0.41$). These results support the assumption of unchanged inhibitory connectivity in Syt2$^{-/-}$ mice. Taken together, our results suggest that Syt2 acts as a fusion clamp at GABAergic BC-PC synapses (Giraudo et al., 2006).

Adenoviral Rescue with Syt2 Generates Fast Transmitter Release

What is the functional significance of the selective usage of Syt2 as a Ca^{2+} sensor at cerebellar BC-PC synapses? Previous studies suggested that different synaptotagmins might differ in their activation and deactivation kinetics (Hui et al., 2005; Xu et al., 2007). However, it is controversial whether Syt2, the isoform expressed in BC terminals, is faster than the other Ca^{2+} sensors (Nagy et al., 2006; Xu et al., 2007; Kochubey et al., 2016). To address this question, we attempted to rescue transmitter release at BC-PC synapses in Syt2$^{-/-}$ mice by viral expression of Syt2, the naturally expressed synaptotagmin isoform (Figures 5 and S3; Table S2). A helper-dependent adenovirus (HdAd) construct was used to express Syt2 and EGFP under the control of two synapsin promoters (Figure 5A). The virus was injected at postnatal day (P) 3 to 6, giving sufficient time for expression until P14–16 (Figure 5B). HdAd-mediated expression of Syt2 in Syt2$^{-/-}$ mice led to a complete rescue of IPSC peak amplitude (Syt2 rescue versus Syt2$^{-/-}$: $p < 0.0001$; Syt2 rescue versus Syt2$^{+/+}$: $p = 0.412$; Figures S3A and S3B). Similarly, adenovirus-mediated expression of Syt2 led to a complete rescue of all other measured synaptic parameters (Figures S3C–3F).

Next, we attempted to rescue transmitter release in Syt2$^{-/-}$ mice by viral expression of Syt1, a synaptotagmin isoform naturally absent from BC-PC synapses (Figures 5 and S3; Table S2). Similar to virally expressed Syt2, HdAd-expressed Syt1 fully rescued IPSC peak amplitude (Syt1 rescue versus Syt2$^{+/+}$; $p = 0.861$). Analysis of EGFP expression and synaptotagmin immunoreactivity in infected BCs suggested that the expression levels for HdAd-Syt2 and HdAd-Syt1 were similar (Figure S2). These results indicate that Syt2 and Syt1 fully rescued the IPSC amplitude in an interchangeable manner.

To directly test whether Syt1 and Syt2 mediated transmitter release with different time course, we quantified the time course of release (TCR) using deconvolution (Figures 5C–5F). Unitary IPSCs were first recorded in standard extracellular solution containing 2 mM Ca^{2+}, aligned to the peak of the presynaptic AP, and averaged to generate a unitary IPSC waveform (Figures 5C and 5D). Subsequently, IPSCs were recorded in extracellular solution containing 0.7 mM Ca^{2+}, aligned to the 50% rise point, and averaged to generate a quantal IPSC waveform (Figure 5D, insets). Finally, the TCR was obtained by deconvolution of the two traces (Experimental Procedures; Figure 5E). For rescue with Syt2, the latency of the TCR was 1.17 ± 0.12 ms and the half-duration was 0.78 ± 0.13 ms (11 pairs). In contrast, for rescue with Syt1, both the latency of the TCR (1.64 ± 0.11 ms) and the half-duration were significantly longer (2.00 ± 0.35 ms; 14 pairs; $p = 0.009$ and 0.006, respectively; Figure 5F). These results indicate that different synaptotagmin isoforms, when expressed in GABAergic synapses in their natural context, have different kinetic properties. Syt2-mediated release shows both a shorter latency and a higher temporal precision.

Rescue with Syt2 Generates Faster Pool Refilling Rates

A hallmark property of GABAergic synapses is the ability to release transmitter in a sustained manner during repetitive stimulation (Kraushaar and Jonas, 2000; Sakaba, 2008; Hu et al., 2014). To test whether the synaptotagmin isoform also controls the rate of replenishment of the vesicular pool, we applied 100-Hz trains of 50 APs and analyzed the dynamics of synaptic transmission after rescue with either Syt2 or Syt1 (Figure 6). Both HdAd-Syt2 and HdAd-Syt1 synapses showed depression during high-frequency trains of stimuli (Figure 6A). However, the ratio $IPSC_{50}/IPSC_1$ was significantly larger for Syt2 rescue than for Syt1 rescue ($IPSC_{50}/IPSC_1$ = 0.56 ± 0.09 for HdAd-Syt2 synapses; 0.21 ± 0.02 for HdAd-Syt1 synapses; ten and nine pairs, respectively; $p = 0.009$). In contrast, synaptic depression after Syt2 rescue was not significantly different from that in Syt2$^{+/+}$ synapses ($IPSC_{50}/IPSC_1$ = 0.46 ± 0.05; nine pairs; $p = 0.54$; Figure 6C). Thus, Syt2 better supported sustained synaptic transmission during high-frequency activity than Syt1.

Differences in the steady-state amplitude of IPSCs during repetitive stimulation could be generated by differences in readily releasable pool size (RRP), release probability, or replenishment rate. We therefore determined these parameters by analysis of cumulative release (Neher, 2015; Figure 6D). To determine absolute values of RRP size and refilling rate, we further measured quantal size by nonstationary fluctuation analysis (Figure S4). Cumulative IPSC amplitude was plotted against stimulus number, and the last ten data points were fit by linear regression. The size of the RRP was then determined from the intersection of the regression line with the ordinate, and release probability was quantified as the ratio $IPSC_1$/RRP size. Finally, the replenishment rate was measured as the slope of the regression line (Figure 6D). Comparison of HdAd-Syt2 and HdAd-Syt1 synapses revealed that the RRP size and release probability were not significantly different (RRP = 44.3 ± 7.1 vesicles and 37.6 ± 5.3 vesicles; P_r = 0.20 ± 0.02 and 0.22 ± 0.03; ten and nine cells; $p > 0.99$ and $p = 0.92$, respectively; Figures 6F, right, and 6G, left). In contrast, the refilling rate was significantly larger in HdAd-Syt2 than HdAd-Syt1 synapses (3.91 ± 0.66 quanta ms^{-1} and 1.78 ± 0.12 quanta ms^{-1}; $p = 0.008$). Refilling rate of Syt2$^{+/+}$ synapses was not significantly different from Syt2 rescue, but markedly different from Syt1 rescue ($p = 0.55$ and 0.006, respectively; Figure 6G, right). Control experiments in the presence of 300 μM of the low-affinity competitive $GABA_A$ receptor antagonist (1,2,5,6-tetrahydropyridin-4-yl)-methylphosphinic acid

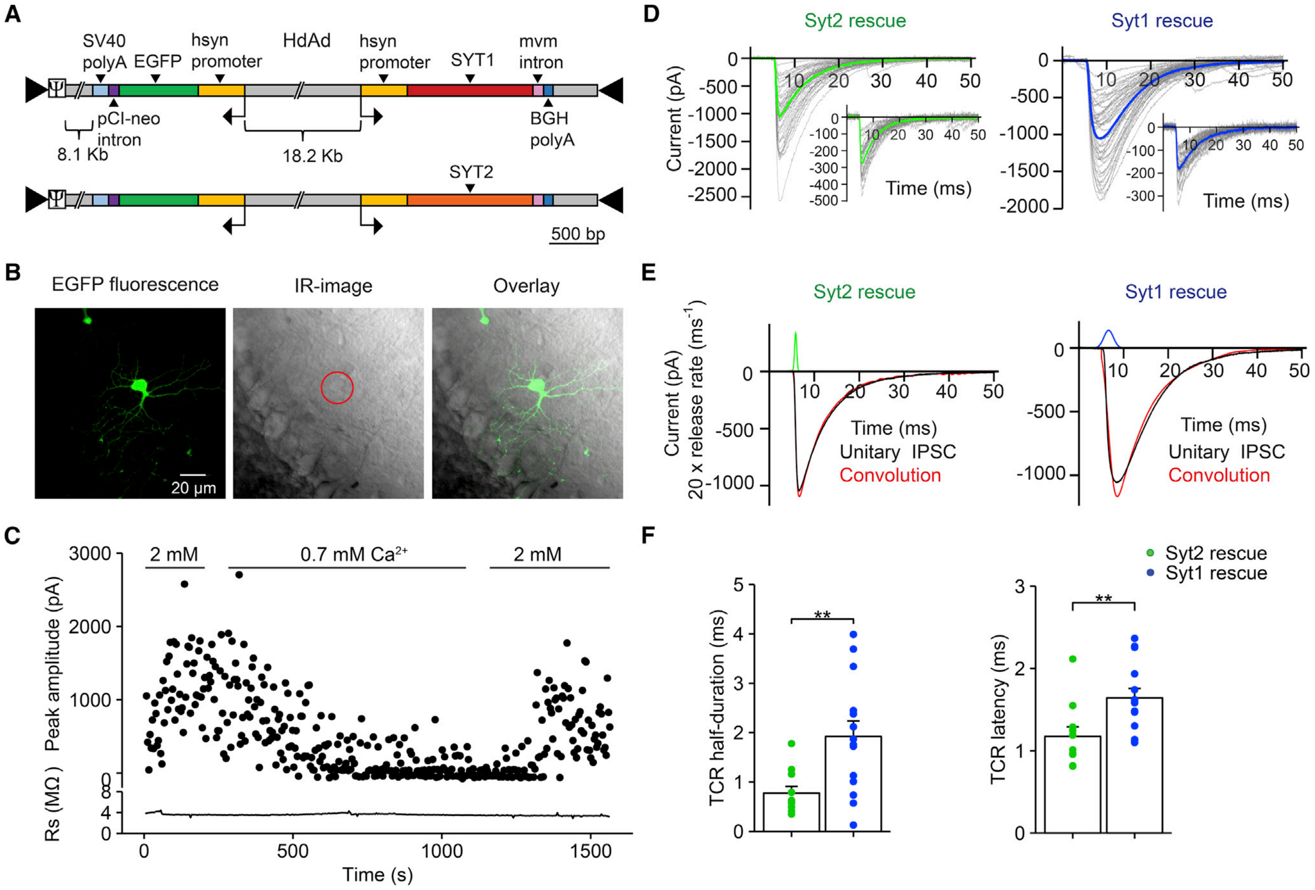

Figure 5. Syt2 Mediates Faster Release Than Syt1 at BC-PC Synapses

(A) Schematic illustration of the helper-dependent adenovirus (HdAd) constructs used for rescue experiments. Hsyn, human synapsin promoter; mvm, minute virus of mice intron; SV40 polyA, Simian virus 40 poly A; BGH polyA, bovine growth hormone poly A.

(B) Confocal stack projection of a cerebellar BC in the molecular layer (left), infrared videomicroscopy light-micrograph (center), and overlay (right). The BC is strongly fluorescent, showing successful infection with HdAd.

(C and D) Analysis of TCR by deconvolution. To acquire unitary IPSCs evoked by single APs, recording was started in an extracellular solution containing 2 mM Ca^{2+}. To isolate quantal IPSCs, recording was continued in an extracellular solution containing 0.7 mM Ca^{2+} (C). Unitary IPSCs were aligned to the peak of the presynaptic AP and averaged (D, main graphs), and quantal IPSCs were aligned to the 50% onset point and averaged (D, insets). Left, HdAd-Syt2 rescue; right, HdAd-Syt1 rescue. Gray lines, individual traces; green and blue lines, averages.

(E) Different TCR after rescue with Syt2 and Syt1. Black, average unitary IPSC; green and blue, TCR (scaled up by a factor of 20); red, results from deconvolution. Left, HdAd-Syt2 rescue; right, HdAd-Syt1 rescue. Data from (C), (D, right), and (E, right) were taken from the same experiment; data from (D, left) and (E, left) were from another experiment.

(F) Summary bar graph of latency (left) and half-duration of TCR (right). Bars represent mean ± SEM; points indicate data from individual experiments. Data were obtained from 11 pairs for HdAd-Syt2 rescue and 14 pairs for HdAd-Syt1 rescue.

(TPMPA) and the $GABA_B$ receptor antagonist CGP55845 (both Tocris) gave similar results (Figure S5), indicating that the results of deconvolution analysis were not confounded by desensitization or saturation of postsynaptic receptors (Jones et al., 2001; Sakaba, 2008; Arai and Jonas, 2014).

To further test whether the synaptotagmin isoform also determined the kinetics of pool refilling after a depleting train, we examined the time course of recovery from depression (Figures 6B, 6E, and 6H). 100-Hz train of 50 stimuli was applied to deplete the pool, followed by a single stimulus to probe the time course of refilling of the pool. Recovery from depression was faster in Syt2-rescued than in Syt1-rescued synapses (Figures 6B and 6E). On average, the recovery time constant was 2.17 ± 0.31 s for HdAd-Syt2 synapses and 4.89 ± 0.79 s for HdAd-Syt1 synapses (13 and 12 pairs, respectively; $p = 0.011$; Figure 6H, left). Similarly, the corresponding replenishment rates were significantly larger for Syt2-rescued than in Syt1-rescued synapses ($p = 0.011$; Figure 6H, right). Recovery time constant and replenishment rate of $Syt2^{+/+}$ synapses were not significantly different from Syt2 rescue, but markedly different from Syt1 rescue ($p = 0.5$ and 0.02, respectively; Figure 6H). In conclusion, the synaptotagmin isoform not only controls the TCR, but also the rate of replenishment of the releasable pool, with faster refilling for Syt2, but slower refilling for Syt1.

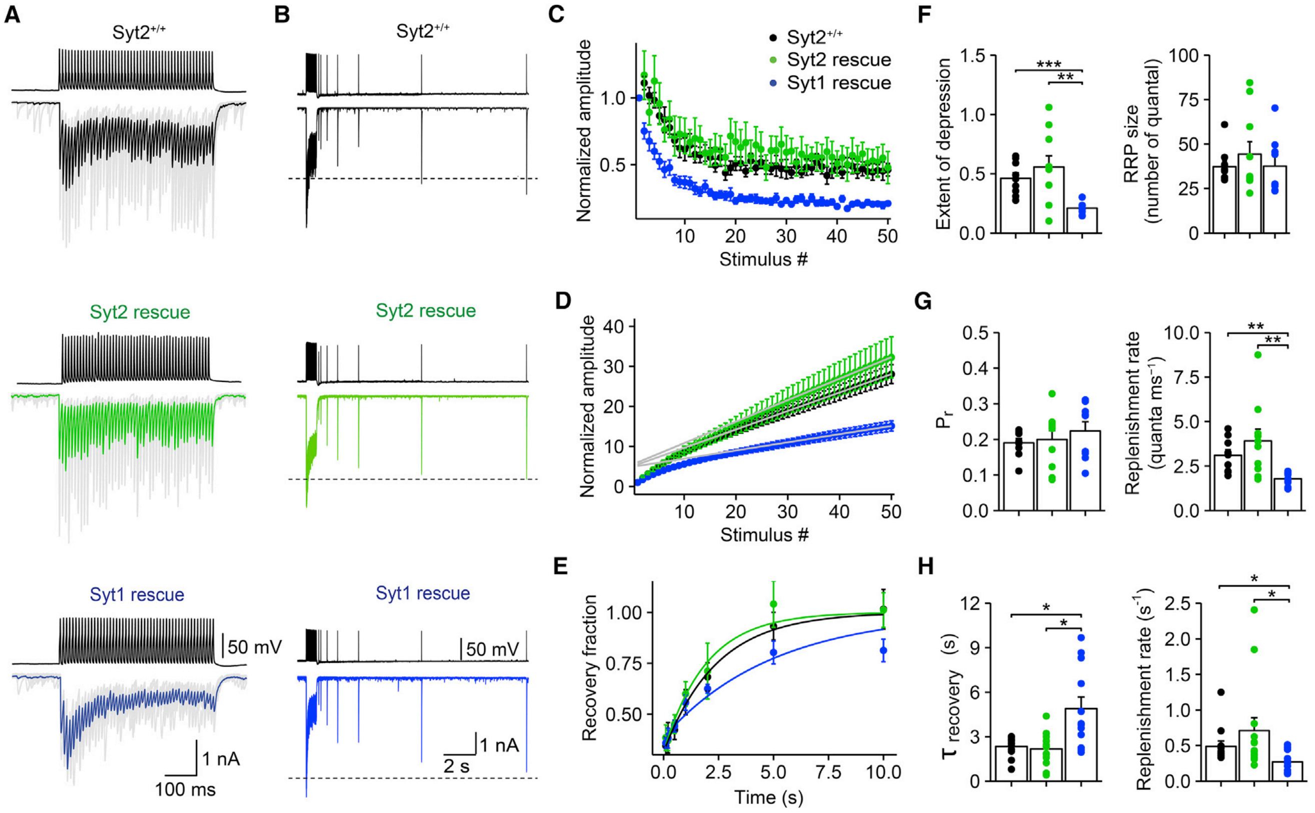

Figure 6. Syt2 Mediates Faster Vesicular Pool Refilling Than Syt1 at BC-PC Synapses

(A and B) Unitary IPSCs evoked by a train of 50 APs at 100 Hz (A) and unitary IPSCs evoked by a train of 50 APs at 100 Hz, followed by a single test stimulus with different recovery time intervals (B), for $Syt2^{+/+}$ (top), HdAd-Syt2 rescue (center) and HdAd-Syt1 rescue (bottom; both on $Syt2^{-/-}$ background). In (A), gray traces represent individual sweeps; black, green, and blue lines represent averages. In (B), traces represent piecewise averages from 35 individual sweeps total; horizontal dashed lines represent $IPSC_1$.

(C) Normalized IPSC peak amplitudes, plotted against stimulus number. Black circles, $Syt2^{+/+}$; green circles, HdAd-Syt2 rescue; blue circles, HdAd-Syt1 rescue (both on $Syt2^{-/-}$ background). Data were obtained from nine, ten, and nine pairs.

(D) Quantitative analysis of pool size and refilling rate. IPSC peak amplitude was divided by $IPSC_1$, averaged across cells, and cumulatively plotted against stimulus number. The last ten points were fit by linear regression. Size of the RRP was determined from intersection of the regression line with the ordinate, whereas refilling rate was determined from the slope of the line. Release probability was quantified as the ratio of $IPSC_1$ over pool size.

(E) Plot of peak amplitude of IPSC evoked by the test stimulus, normalized to the amplitude of the first IPSC in the preceding train. Continuous curves represent exponential functions fit to the data points. Data were obtained from 12, 13, and 12 pairs. Error bars indicate SEM.

(F–H) Summary bar graph of steady-state depression ($IPSC_{50}/IPSC_1$; F, left), RRP (F, right), release probability (P_r, G, left), replenishment rate during train (G, right), recovery time constant (H, left), and replenishment after train (H, right).

Black, data from $Syt2^{+/+}$ synapses; green, HdAd-Syt2 rescue; blue, HdAd-Syt1 rescue (both on $Syt2^{-/-}$ background). Bars represent mean ± SEM; circles indicate data from individual experiments.

Syt2 Is Essential for Feedforward Inhibition in Cerebellum

The present results suggest that the selective use of Syt2 at BC synapses plays a key role for the efficacy and timing of inhibitory synaptic transmission in the cerebellum, parameters, which are highly relevant for feedforward inhibition. To directly test the role of Syt2 in feedforward inhibition, we measured amplitude and timing of disynaptic IPSCs evoked by extracellular parallel fiber stimulation (Mittmann et al., 2005; Bao et al., 2010; Figure 7). Monosynaptic EPSCs and disynaptic IPSCs were either measured together at a holding potential of −50 mV, where EPSCs were detected as inward currents and IPSCs as outward currents (Figure 7B), or at membrane potentials of −60 and 0 mV, where EPSCs and IPSCs could be studied in isolation (Figures 7C and S6). In slices from $Syt2^{+/+}$ mice, monosynaptic EPSCs were followed by large disynaptic IPSCs, confirming powerful feedforward inhibition in this circuit (Mittmann et al., 2005). In contrast, in slices from $Syt2^{-/-}$ mice, disynaptic IPSCs were markedly impaired (Figures 7B and 7C). Comparison of slices from $Syt2^{+/+}$ and $Syt2^{-/-}$ mice indicated that the excitatory peak conductance was the same (2.3 ± 0.4 versus 2.7 ± 0.6 ns; ten and nine cells; $p = 0.84$), whereas the inhibitory peak conductance was severely reduced (14.9 ± 3.7 versus 0.7 ± 0.2 ns; $p < 0.001$). Furthermore, the delay between EPSCs and IPSCs was markedly prolonged (4.9 ± 0.6 versus 20.8 ± 6.4 ms; $p < 0.001$). These results demonstrate that Syt2 plays a critical role for both efficacy and timing of feedforward inhibition in the cerebellum.

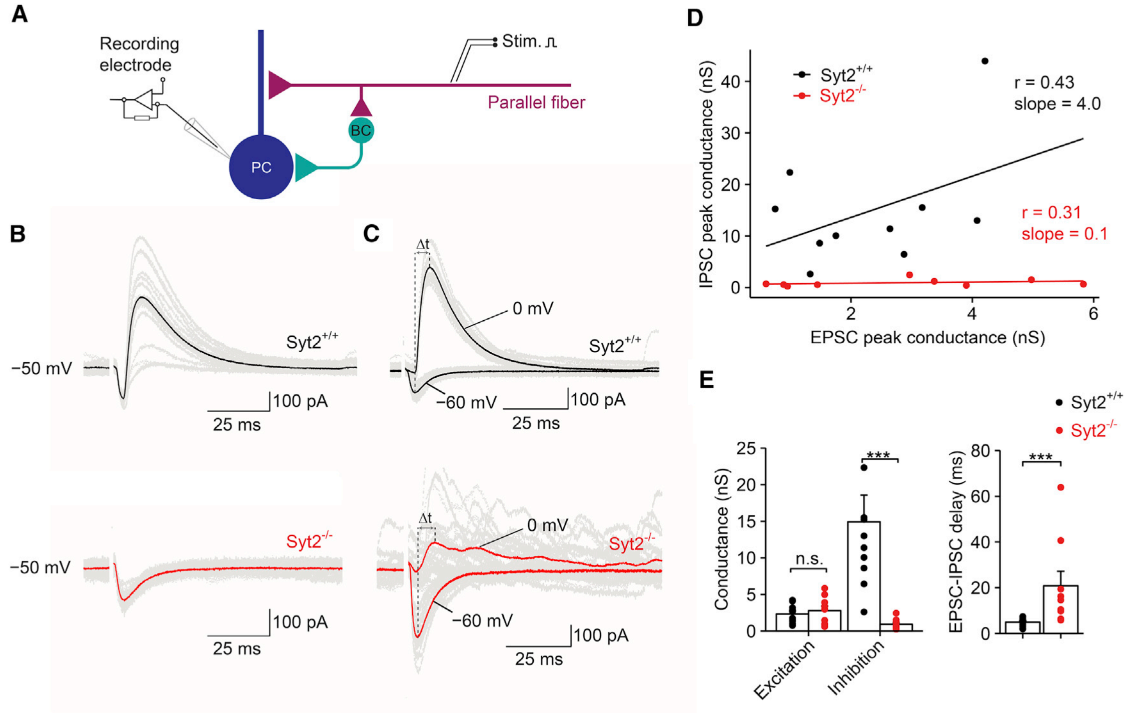

Figure 7. Fast Feedforward Inhibition in Cerebellum Critically Depends on the Presence of Syt2

(A) Schematic illustration of recording configuration for analysis of feedforward inhibition. Whole-cell recording from PC; stimulation of parallel fibers is expected to evoke both monosynaptic EPSCs and disynaptic IPSCs.

(B) Recording of mixed EPSCs and IPSCs at −50 mV. As the holding potential is between the reversal potentials for excitatory and inhibitory events, EPSCs are inwardly and IPSCs outwardly directed.

(C) Recording of EPSCs and IPSCs isolated by setting the holding potential to the reversal potential of one of the conductances (−60 mV and 0 mV, respectively). Top, $Syt2^{+/+}$; bottom, $Syt2^{-/-}$. Single traces (gray) and averages (black for $Syt2^{+/+}$ and red for $Syt2^{-/-}$).

(D) Scatterplot of inhibitory (IPSG) against excitatory peak conductance (EPSG) for $Syt2^{+/+}$ (black) and $Syt2^{-/-}$ synapses (red). Data points were fit by linear regression. Similar extracellular stimulus intensities in the two datasets.

(E) Summary bar graphs of peak conductance (left) and EPSC–IPSC delay (measured from peak to peak). Bars represent mean ± SEM; circles indicate data from individual experiments.

DISCUSSION

The present results provide insights into the mechanisms of Ca^{2+}-dependent exocytosis at the cerebellar BC-PC synapse, a major inhibitory synapse in the brain. First, we identified Syt2 as the primary Ca^{2+} sensor of exocytosis. Second, viral rescue experiments revealed that the naturally occurring sensor Syt2 mediated transmitter release with shorter latency and higher temporal precision than the alternative sensor Syt1. Finally, Syt2 mediated faster refilling of the vesicular pool during repetitive stimulation than Syt1, suggesting that Syt2 controls both exo- and endocytosis at GABAergic synapses. Thus, the use of Syt2 as a release sensor contributes to rapid signaling at this GABAergic synapse (Figure S7).

Syt2-Mediated Fast GABA Release at Inhibitory Synapses

The mammalian genome encodes 16 synaptotagmins, eight of which bind Ca^{2+} (Syt1, 2, 3, 5, 6, 7, 9, and 10; Chapman, 2002; Südhof, 2002), and three of which were reported to act as fast release sensors (Syt1, 2, and 9; Xu et al., 2007; Kochubey et al., 2016). However, the functional significance of this molecular diversity is incompletely understood.

It is generally thought that Syt1 is predominant in cortical circuits, whereas Syt2 is more prevalent in the cerebellum, brainstem, and spinal cord (Pang et al., 2006a, 2006b; Mittelsteadt et al., 2009; Kochubey et al., 2016). However, our immunohistochemical analysis showed that Syt1 is highly expressed in the cerebellum, where it is mainly localized to excitatory synapses. This idea is supported by the disynaptic inhibition experiments, which show that Syt2 deletion suppresses inhibition but leaves excitation unchanged (Figure 7). Thus, Syt1 and Syt2 coexist in the circuit but are expressed in a synapse-specific manner.

It is also believed that Syt2 is more abundantly used in GABAergic interneurons. Consistent with this idea, Syt2 is highly expressed in subsets of GABAergic hippocampal interneurons (Pang et al., 2006a; Kerr et al., 2008; Sommeijer and Levelt, 2012). However, genetic deletion of Syt1 substantially reduces, but does not abolish, transmitter release (Kerr et al., 2008), suggesting that in the hippocampus Syt1 and Syt2 may work in concert. The results from immunocytochemistry, knockout, and rescue experiments convergently suggest that Syt2 is the major

Ca^{2+} sensor triggering fast transmitter release at the cerebellar BC-PC synapse. Thus, both cell type (GABAergic interneurons versus glutamatergic principal neurons) and brain region (cerebellum versus other brain areas) determine which synaptotagmin isoform is used.

The molecular identity of the Ca^{2+} sensor mediating the residual release component in $Syt2^{-/-}$ synapses is presently unclear. The residual component might be generated by a compensatory upregulation of Syt1, although our immunolabeling data argue against this possibility (Figures S1C and S1D). Alternatively, any of the other synaptotagmins, or an entirely different Ca^{2+} sensor, may be involved. One possibility is that the residual component is mediated by Syt7 (Bacaj et al., 2013; Jackman et al., 2016). Consistent with this idea, the residual component shows profound facilitation and highly asynchronous kinetics (Figure 3). Analysis of double-knockout mice will be needed to test this hypothesis.

Syt2 Controls Fast Exo- and Endocytosis

The BC-PC synapse provides an ideal system to compare the functional properties of Syt2 with those of Syt1, the alternative Ca^{2+} sensor in synaptic transmission. As fast transmitter release at this synapse is almost completely dependent on Syt2, rescue experiments can be performed in $Syt2^{-/-}$ animals with minimal confounding effects of other synaptotagmin isoforms. Our rescue experiments revealed that Syt2 differs from Syt1 in both speed and temporal precision of transmitter release. Thus, our results corroborate the original suggestion of higher temporal precision for Syt2-mediated release (Xu et al., 2007) and extend these findings by showing that the synaptic latency is also isoform dependent. However, they appear to be inconsistent with a recent study, which found no kinetic differences between Syt1 and Syt2 at the calyx of Held (Kochubey et al., 2016).

Differences in the shape of the Ca^{2+} transient at the sensor may explain the apparent discrepancies. In the present study, release was evoked by natural AP waveforms. Because of the tight coupling between Ca^{2+} channels and sensors (Arai and Jonas, 2014), the Ca^{2+} transient "seen" by the sensor will be short, closely following the presynaptic Ca^{2+} current. Thus, the TCR will be shaped by the activation and deactivation rates of the sensor. In contrast, in the study by Kochubey et al. (2016), release was triggered by long voltage pulses. Therefore, the rise in Ca^{2+} concentration will be long lasting, and the TCR will be primarily shaped by the pool depletion and refilling; the deactivation rates of the sensor may be less relevant.

In addition to the difference in TCR, we found that synaptotagmins differentially controlled the rate of refilling of the releasable pool. Whereas the size of the RRP and the probability of release from this pool were similar for Syt2 and Syt1, the refilling rate was $\sim$2-fold faster for Syt2 rescue. This result is consistent with several previous observations: that synaptotagmins couple to AP2/clathrin (Zhang et al., 1994), that endocytosis at the *Drosophila* neuromuscular junction is suppressed by light inactivation of synaptotagmin (Poskanzer et al., 2003) and that endocytosis at the calyx of Held is blocked by AP2 peptides (Hosoi et al., 2009). Thus, Syt2 may control both the speed of GABA release following single APs and the efficacy of release during trains of APs. Previous studies showed that the replenishment of the RRP at BC-PC synapses is dependent on intracellular Ca^{2+} concentration (Sakaba, 2008). Our results are consistent with the hypothesis that Syt2 is the molecular sensor that mediates the Ca^{2+} dependence of replenishment. A caveat of the rescue experiments is that differences in expression levels between Syt1 and Syt2 cannot be entirely excluded (Experimental Procedures). Whether such differences affect the time course of exocytosis and endocytosis remains to be determined.

A Clamping Function of Syt2 at GABAergic Synapses?

Whether genetic elimination of synaptotagmins increases the frequency of spontaneous release has been controversial. One potential problem is that changes in miniature release may be confounded by sprouting or homeostatic changes. Furthermore, the effects of synaptotagmin deletion on spontaneous release depend on the synaptic environment (Liu et al., 2009). Our results rigorously address this question. First, analysis of synaptic transmission is possible in the intact circuit, because of the extended survival of $Syt2^{-/-}$ mice in comparison to, e.g., $Syt1^{-/-}$ mice (Geppert et al., 1994; Kerr et al., 2008). Second, immunolabeling experiments reveal that the organization of the inhibitory microcircuits is maintained in the $Syt2^{-/-}$ mice (Figures 4E and 4F). Taken together, these results are consistent with a clamping function of Syt2 at BC-PC synapses (Giraudo et al., 2006). The molecular mechanisms underlying this clamping function remain to be determined. Clamping could be achieved by an arrest of the partially zippered SNARE complex (Chicka et al., 2008). Alternatively, clamping may be generated by the competition of synaptotagmins for binding sites in the release machinery. In this model, Syt2 may prevent the access of other synaptotagmin isoforms, which may drive release at lower Ca^{2+} concentrations or even in the absence of Ca^{2+}.

Whether synaptotagmin "clamps" asynchronous release also has remained unclear. Genetic elimination of Syt1 at glutamatergic synapses was shown to selectively eliminate synchronous release, while asynchronous release was either unaffected (Geppert et al., 1994) or enhanced (Nishiki and Augustine, 2004). Differential effects on asynchronous release during and after a stimulus train have been also suggested (Maximov and Südhof, 2005). Our results show a significant enhancement of asynchronous release both during and after the train (Figure 3). This is consistent with a dual function of Syt2, which acts as both a trigger of synchronous release and a clamp of asynchronous release. Alternatively, it was proposed that synaptotagmins may operate as pure synchronizers of release (Nishiki and Augustine, 2004). However, for a pure synchronizer, the reduction in synchronous release should equate the enhancement of asynchronous release, which is not the case at BC-PC synapses. Thus, our results for Syt2 at GABAergic synapses seem inconsistent with a pure synchronizing function.

Molecular Mechanisms Underlying Differential Kinetics

Our results demonstrate that Syt2 has a kinetic advantage in terms of speed and temporal precision of synaptic transmission. What are the underlying molecular mechanisms? Syt2 has a sequence identity of $\sim$60% with Syt1 in mice (Südhof, 2002). The C2A domain is largely conserved between Syt2 and Syt1, with only one amino acid difference in the three loops

forming the putative Ca^{2+} binding site. However, the C2B domain is more divergent between isoforms, with three amino acid differences in the relevant loops (Südhof, 2002). These structural differences might explain our observations for two reasons. First, the C2B domain seems more relevant for the exocytotic Ca^{2+} sensing function than the C2A domain (Mackler et al., 2002; Nishiki and Augustine, 2004; Bacaj et al., 2013). Second, the C2B domain is thought to represent the binding site for AP2, which might explain the effects of synaptotagmin isoform on pool replenishment.

Other regions of the synaptotagmin molecule may be also important. For example, the linker between C2A and C2B domains shows three amino acid differences between Syt1 and Syt2. Recent work suggested this linker to be critical for the function of synaptotagmins (Liu et al., 2014). In this scenario, the presence of two glycine residues in Syt2 could make the linker more flexible. Furthermore, the connector between the transmembrane segment and the C2A domain is seven amino acids shorter for Syt2 than for Syt1. This could be relevant for tight coupling between Ca^{2+} channels and synaptotagmins (Eggermann et al., 2011). Consistent with this idea, proteomic analysis revealed that Syt2, but not Syt1, is molecularly associated with Ca^{2+} channel alpha subunits (Müller et al., 2010). Finally, the C terminus of synaptotagmins is highly divergent between the isoforms (Young and Neher, 2009). The C terminus has been also suggested to be important for coupling but is also for internalization of the protein from the plasma membrane (Jarousse and Kelly, 2001). Thus, both coupling distance and rate of endocytosis might be regulated by this region. Differential binding of Syt isoforms to Ca^{2+} channels or other presynaptic ion channels may also modify channel gating, and thereby affect the time course of presynaptic Ca^{2+} current, AP waveform, or both. Direct recordings from inhibitory presynaptic terminals will be needed to address these possibilities.

Relevance for Microcircuit Function

The selective use of Syt2 at BC output synapses may have important consequences for the function of cerebellar microcircuits. First, Syt2 may control the speed of feedforward inhibition (Mittmann et al., 2005; Bao et al., 2010). One major function of feedforward inhibition is that it narrows the time window for spiking and temporal summation (Pouille and Scanziani, 2001; Mittmann et al., 2005). Another function is shaping of the spatial activity pattern of PC activation following APs in parallel fiber beams (Mittmann et al., 2005; Bao et al., 2010). For both functions, the fast time course of inhibition is critically important. Previous studies showed that the delay of disynaptic inhibition is as short as ~1 ms at physiological temperature (Mittmann et al., 2005). We identify Syt2 as a molecular factor that contributes to this remarkable speed. Both BCs and stellate cells are thought to contribute to disynaptic inhibition. The sensitivity of disynaptic IPSCs to Syt2 deletion (Figure 7) suggests that both BC and stellate cell output synapses use Syt2 for transmitter release. Paired recording experiments will be required to directly determine the identity of the sensor at stellate cell synapses.

Second, the selective use of Syt2 may be relevant for sustained inhibition in the intact network in vivo. In the awake, behaving animal, molecular layer interneurons fire APs at a frequency of ~20 Hz under resting conditions, and can be further activated by sensory stimuli (Ekerot and Jörntell, 2003). Under these conditions, depletion of the releasable pool would be expected. Therefore, the rapid replenishment of the pool mediated by Syt2 helps to maintain inhibitory output.

Finally, Syt2 may be important in synaptic diseases. In $Syt2^{-/-}$ or Syt2 mutant mice, the ataxia phenotype (Pang et al., 2006a, 2006b) is readily explained by a failure of feedforward inhibition. Mutations in the Syt2 gene have been recently identified in humans (Herrmann et al., 2014). These mutations were primarily linked to a neuromuscular phenotype (Herrmann et al., 2014). Whether other mutations in the Syt2 gene do exist and whether these may generate a cerebellar phenotype, as predicted by the present data, remains to be determined.

EXPERIMENTAL PROCEDURES

Animal Experiments

Experiments on C57BL/6 wild-type and mutant mice were performed in strict accordance with institutional, national, and European guidelines for animal experimentation and were approved by the Bundesministerium für Wissenschaft, Forschung, und Wirtschaft of Austria (A. Haslinger, Vienna; BMWF-66.018/0008-II/3b/2010; BMWF-66.018/0010-WF/V/3b/2015).

Immunohistochemistry

Brains of 14- to 16-day-old mice were dissected out, fixed in 4% paraformaldehyde and 1% sucrose for ~2 hr, and transferred to 30% sucrose in PBS (~10 hr) for cryoprotection. 50-μm-thick slices were cut from the cerebellar vermis using a cryostat (HM560; Thermo Scientific). After washing with 0.01 M PBS, slices were incubated with 10% normal goat serum (NGS) for 1 hr and subsequently with primary monoclonal antibodies against Syt1 (immunoglobulin G2b [IgG2b], mab48, 1:500, developmental studies hybridoma bank, DSHB), Syt2 (IgG2a, znp1, 1:500, zebrafish international resource center, ZIRC; Fox and Sanes, 2007), or both in PBS containing 5% NGS and 0.3% Triton X-100 overnight. After washing, slices were incubated with isotype-specific secondary antibodies (goat anti-mouse IgG2b for mab48 and goat anti-mouse IgG2a for znp1, 1:1000 for both; Invitrogen) with PBS containing 3% NGS and 0.3% Triton X-100 overnight. After washing, slices were embedded in Prolong Antifade and examined under a TCS SP5 II confocal microscope (Leica Microsystems). Syt2 immunolabeling was completely absent in $Syt2^{-/-}$ mice (Figure S1C). For double labeling for VGAT or VGLUT1, rabbit polyclonal antibodies (#131003 and #135303; Synaptic Systems) were used.

Confocal stacks were analyzed using the open-source software Fiji ("Fiji is just ImageJ"). For analyzing puncta in different layers of the cerebellum, regions of interest were randomly defined using the "Freehand Selection" feature of Fiji. Threshold was automatically adjusted using the "Triangle" method for both channels. Puncta number and area was analyzed using the "Analyze Particles" function.

Cerebellar Slice Preparation

C57BL/6 Syt2 knockout mice ($Syt2^{-/-}$), in which exons 2–7 of the Syt2 gene were deleted, were kindly provided by T.C. Südhof, Stanford University (Pang et al., 2006a). All experiments were performed on littermate offspring from heterozygous matings, with knockout mice being homozygous for the deletion allele ($Syt2^{-/-}$) and wild-type animals homozygous for the wild-type allele ($Syt2^{+/+}$). Slices were cut from the cerebellum of 14- to 16-day-old mice of either sex. In all experiments, genotypes were determined by PCR analysis. After decapitation, the brain was rapidly dissected out and immersed in ice-cold slicing solution containing: 87 mM NaCl, 25 mM $NaHCO_3$, 2.5 mM KCl, 1.25 mM NaH_2PO_4, 10 mM D-glucose, 75 mM sucrose, 0.5 mM $CaCl_2$, and 7 mM $MgCl_2$ (pH 7.4 in 95% O_2/5% CO_2, 325 mOsm). Parasagittal 250-μm-thick cerebellar slices from the vermis region were cut using a custom-built or a VT1200 vibratome (Leica Microsystems). After ~20-min incubation at

~35°C, the slices were stored at room temperature. Slices were used for maximally 5 hr after dissection. Experiments were performed at 21°C–24°C.

Electrophysiology

During experiments, slices were superfused with a physiological extracellular solution containing: 125 mM NaCl, 2.5 mM KCl, 25 mM $NaHCO_3$, 1.25 mM NaH_2PO_4, 25 mM D-glucose, 2 mM $CaCl_2$, and 1 mM $MgCl_2$ (pH 7.4 in 95% O_2/5% CO_2, ~325 mOsm). Paired recordings from synaptically connected BCs and PCs were performed as described previously (Caillard et al., 2000; Sakaba, 2008; Christie et al., 2011; Eggermann and Jonas, 2011; Arai and Jonas, 2014). Intracellular solution used for the presynaptic BCs contained: 125 mM K-gluconate, 20 mM KCl, 0.1 mM EGTA, 10 mM phosphocreatine, 2 mM $MgCl_2$, 2 mM ATP, 0.4 mM GTP, 10 mM HEPES (pH adjusted to 7.28 with KOH, ~310 mOsm); 0.2% biocytin was added in a subset of recordings. The presynaptic pipette resistance was 8–15 MΩ. BCs were recorded under current-clamp conditions. A holding current of approximately −50 pA was injected to maintain the membrane potential at approximately −65 mV and to avoid spontaneous AP generation. To evoke presynaptic APs, single pulses or trains of either ten pulses at 50 Hz or 50 pulses at 100 Hz (400 pA, 4 ms) were injected into the presynaptic BC every 4 or 20 s, respectively.

Intracellular solution for postsynaptic PCs contained: 140 mM KCl, 10 mM EGTA, 2 mM $MgCl_2$, 2 mM ATP, 10 mM HEPES, and 2 mM QX-314 (pH adjusted to 7.28 with KOH, ~313 mOsm). To achieve the lowest possible postsynaptic series resistance, leaded glass (PG10165-4, World Precision Instruments [WPI]) was used to fabricate large tip-sized recording pipettes. The postsynaptic pipette resistance was 0.8–1.5 MΩ, resulting in a series resistance of 3–8 MΩ. Experiments in which series resistance changed by >2 MΩ were discarded. PCs were recorded in the voltage-clamp configuration with a holding potential of −70 mV. For monitoring series and input resistance, 5-mV, 100-ms hyperpolarizing test pulses were applied after the IPSCs had decayed to baseline.

For recording of mIPSCs (Figure 4), synaptic events were examined in pharmacological isolation in the presence of 1 μM TTX, 10 μM CNQX, and 20 μM D-AP5 at −70 mV. For feedforward inhibition experiments (Figures 7 and S6), slices were cut in frontal plane to preserve parallel fibers (Mittmann et al., 2005). Parallel fibers were stimulated with glass pipettes (~1 MΩ) containing extracellular solution placed at a distance >100 μm from the recorded PC to avoid direct stimulation of interneuron axons. The stimulus electrode was placed in the center or outer half of the molecular layer. Electrical stimuli (5–8 V amplitude, 100-μs duration) were delivered using a stimulus isolation unit. PCs were recorded in the whole-cell configuration using patch pipettes similar to those in paired recordings. Membrane potential was set to −50 mV to record both EPSCs and IPSCs in the same trace and to either −60 mV or 0 mV to examine EPSCs and IPSCs in isolation. The electrode solution contained: 130 mM K-methanesulfonate, 2 mM KCl, 10 mM EGTA, 2 mM $MgCl_2$, 2 mM Na_2ATP, 10 mM HEPES, and 5 mM QX-314 (pH adjusted to 7.27 with KOH). In a subset of experiments, the selective $GABA_A$ receptor antagonist SR-95531 (10 μM, Biotrend) was used to block IPSCs.

Production of Adenoviral Expression Vectors

Synaptotagmin cDNA (*Mus musculus* isoforms 1 and 2) was codon-optimized for expression in mouse (GeneArt) and then cloned into the EcoRI and NotI sites of the synapsin expression cassette (Montesinos et al., 2011). This cassette included the 470-bp human synapsin (hsyn) promoter, the minute virus of mice (mvm) intron, and the bovine growth hormone (BGH) polyA. Subsequently, the expression cassette was cloned into the AscI site of pdelta28E4, a gift from Dr. Phil Ng (Palmer and Ng, 2003), using InFusion (Clontech). This version of pdelta28E4 was modified to also contain a separate neurospecific EGFP expression cassette driven by the 470-bp hsyn promoter. The final HdAd plasmids allow for expression of synaptotagmin isoforms independently of EGFP as dual expression recombinant Ad vectors, similar to the strategy previously used with second-generation rAd vectors (Young and Neher, 2009; Chen et al., 2013).

Production of HdAd was carried out as previously described (Palmer and Ng, 2003). Briefly, HdAds were produced by first digesting the pHdAd with PmeI to linearize and expose the ends of the 5′ and 3′ inverted terminal repeats. Transfection of the pHdAd was performed using 116 producer cells, a modified HEK293 line expressing high levels of Cre recombinase, and a 4-kbp adenoviral genome fragment that encodes for the E1A/E1B gene, necessary for rAd to replicate (Palmer and Ng, 2003). Standard protocols for recombinant HdAd were followed (Palmer and Ng, 2003) with slight modifications. HdAd was serially amplified in five consecutive passages from 3- to 60-mm tissue culture dishes followed by 1- to 15-cm and finally 30- to 15-cm dishes. Each successive passage was performed after cytopathic effect (CPE) occurred and cell lysates were subjected to three freeze/thaw cycles to lyse cells and thereby release the viral particles. HdAd was stored at −80°C in storage buffer containing 10 mM HEPES, 250 mM sucrose, and 1 mM $MgCl_2$ at pH 7.4. Viral particle concentration (ml^{-1}) was calculated (Palmer and Ng, 2003) as follows: viral particles/mL = (A260) × (dilution factor) × (1.1 × 10^{12}) × (36) / (size of the vector in kilobases). Virus titers were similar for HdAd-Syt1 and HdAd-Syt2: 4.39 × 10^{12} vp ml^{-1} and 3.98 × 10^{12} vp ml^{-1}, respectively.

Virus Injection

$Syt2^{-/-}$ mice, at postnatal days (P) 3–6, were anesthetized using isoflurane (5% for induction and ~3% during the injection procedure) (Forane, Abbott) combined with meloxicam (1 mg kg^{-1}, Boehringer) for analgesia. Meloxicam was given 2 hr before surgery for pre-operative analgesia and repeated twice 24 hr after the previous injection for post-operative analgesia. After sufficient sedation, mice were put on a stereotaxic apparatus and head-fixed with ear bars. The skin was cut, the skull was exposed, and a small hole was made with a needle in the region over the cerebellum. 1 μL adenovirus (~10^9 vp μL^{-1}) was injected into the vermis of the cerebellum at a depth of ~600 μm from the endocranium. After virus injection, pups were returned to their home cages for recovery. Recordings were made at P14–16. Infected cerebellar BCs were identified by EGFP fluorescence. For electrophysiology, epifluorescence illumination was used. For analysis of rescue efficiency and documentation, cells were examined using a TCS SP5 II confocal microscope (Figure 5B).

To address the expression level after viral infection, we examined both EGFP fluorescence and Syt1 or Syt2 immunoreactivity. Expression levels probed with this approach were very similar for Syt1 and Syt2 (Figure S2). This is consistent with previous results using an identical transgene cassette, demonstrating that Syt1 and Syt2 expression levels probed with a Myc tag and an anti-Myc antibody were comparable (Kochubey et al., 2016).

Data Acquisition and Analysis

Data were acquired with a Multiclamp 700B amplifier (Axon Instruments), low pass filtered at 10 kHz, and sampled at 20 or 50 kHz using a CED power1401 interface (Cambridge Electronic Design). Stimulus generation and data acquisition were performed using custom-made software (FPulse v.3.19 and 3.33, Ulrich Fröbe, University of Freiburg) running under Igor Pro 6.22 (WaveMetrics). Data were analyzed using Stimfit 0.14.9 (https://github.com/neurodroid/stimfit), Igor Pro 6.22, R 3.3.0 (the R project for statistical computing), and Mathematica 10.3 (Wolfram Research). Synaptic latency of monosynaptic IPSCs was measured from the peak of the presynaptic AP to the IPSC onset. Latency of disynaptic IPSCs (Figure 7E) was measured from the peak of the EPSC at −60 mV to that of the IPSC at 0 mV. IPSC decay time constant was determined by fitting the decay phase of an average IPSC trace. To quantify multiple-pulse depression, traces were averaged and the amplitude of each IPSC in a train was measured from the baseline directly preceding the rising phase. mIPSCs were detected using a template matching algorithm and verified by visual inspection (Pernía-Andrade et al., 2012; Goswami et al., 2012). Synchronous and asynchronous release was quantified by a deconvolution algorithm based on Fourier transformation, using trains of ten APs at 50 Hz as stimuli (Hefft and Jonas, 2005). Synchronous release was measured 0–5 ms after each AP. Asynchronous release during the train was quantified 15–20 ms after each AP, and asynchronous release after the train was quantified >20 ms after end of the train. For display purposes, the TCR was filtered at 2 kHz (Figures 3D and 3E).

Latency and half-duration of the TCR following a single AP were quantified by a deconvolution algorithm in which the TCR function was represented by a Gaussian or a gamma distribution. Unitary IPSCs recorded in 2 mM extracellular Ca^{2+} concentration were aligned to the peak of the presynaptic AP and averaged. Quantal IPSCs recorded in 0.7 mM extracellular Ca^{2+} and 2.3 mM

Mg^{2+} were aligned to the 50% rising point and also averaged. The TCR function was convolved with the quantal IPSC waveform, using ListConvolve of Mathematica. Amplitude and shape of the time course of release function were adjusted to minimize the sum of squared differences between predicted and measured unitary IPSC.

For analysis of vesicular pool size and refilling rate, IPSC amplitudes during a 100-Hz train of 50 stimuli were examined. IPSC values were normalized by $IPSC_1$, averaged across cells, and cumulatively plotted against stimulus number. The last ten data points were fit by linear regression. The size of the RRP was determined from intersection of the regression line with the ordinate, whereas refilling rate was determined from the slope (Neher, 2015). The RRP estimate represents "pool decrement" rather than absolute pool size; the true pool size will be larger than the estimate (Neher, 2015). Release probability was quantified as the ratio of normalized $IPSC_1$ over pool size. For obtaining absolute numbers of RRP size and refilling rate, estimated values were multiplied by the quantal content of $IPSC_1$. Quantal size was estimated by nonstationary fluctuation analysis. Variance was plotted against mean for all IPSCs in the train, and analyzed by linear regression. Quantal size was determined from the slope of the fit line (Scheuss et al., 2002; Sakaba, 2008).

Statistics and Conventions

All values were reported as mean ± SEM. Statistical significance was tested using a Kruskal-Wallis and two-sided Wilcoxon rank-sum test in R. Differences with $p < 0.05$ were considered significant. In figures, *$p < 0.05$, **$p < 0.01$, and ***$p < 0.001$, respectively. In experiments with parallel fiber stimulation, stimulation artifacts were blanked for display purposes.

SUPPLEMENTAL INFORMATION

Supplemental Information includes seven figures and two tables and can be found with this article online at http://dx.doi.org/10.1016/j.celrep.2016.12.067.

AUTHOR CONTRIBUTIONS

C.C. and I.A. performed the experiments, C.C., I.A., and P.J. analyzed the data, R.S. and S.M.Y. generated the viruses, and P.J. wrote the paper. All authors jointly revised the paper.

ACKNOWLEDGMENTS

We thank Drs. Nils Brose and Erwin Neher for reading the manuscript. We also thank Z. Pang and T. Südhof for providing Syt2 knockout mice, A. Schlögl for programming, F. Marr for technical assistance, E. Kramberger for manuscript editing, M. Schunn and J. Primus (preclinical facility) for mouse colony management, and E. Papusheva (bioimaging facility) for help with data analysis. This project has received funding from the Austrian Science Fund (FWF) (P 24909-B24) and the European Research Council (ERC) under the seventh framework programme with grant agreement number 268548 (both P.J.) and the Max Planck Society (S.M.Y.).

Received: August 11, 2016
Revised: November 18, 2016
Accepted: December 20, 2016
Published: January 17, 2017

REFERENCES

Arai, I., and Jonas, P. (2014). Nanodomain coupling explains Ca^{2+} independence of transmitter release time course at a fast central synapse. eLife *3*, e04057.

Bacaj, T., Wu, D., Yang, X., Morishita, W., Zhou, P., Xu, W., Malenka, R.C., and Südhof, T.C. (2013). Synaptotagmin-1 and synaptotagmin-7 trigger synchronous and asynchronous phases of neurotransmitter release. Neuron *80*, 947–959.

Bao, J., Reim, K., and Sakaba, T. (2010). Target-dependent feedforward inhibition mediated by short-term synaptic plasticity in the cerebellum. J. Neurosci. *30*, 8171–8179.

Bucurenciu, I., Kulik, A., Schwaller, B., Frotscher, M., and Jonas, P. (2008). Nanodomain coupling between Ca^{2+} channels and Ca^{2+} sensors promotes fast and efficient transmitter release at a cortical GABAergic synapse. Neuron *57*, 536–545.

Caillard, O., Moreno, H., Schwaller, B., Llano, I., Celio, M.R., and Marty, A. (2000). Role of the calcium-binding protein parvalbumin in short-term synaptic plasticity. Proc. Natl. Acad. Sci. USA *97*, 13372–13377.

Chapman, E.R. (2002). Synaptotagmin: A Ca^{2+} sensor that triggers exocytosis? Nat. Rev. Mol. Cell Biol. *3*, 498–508.

Chen, Z., Cooper, B., Kalla, S., Varoqueaux, F., and Young, S.M., Jr. (2013). The Munc13 proteins differentially regulate readily releasable pool dynamics and calcium-dependent recovery at a central synapse. J. Neurosci. *33*, 8336–8351.

Chen, Z., Das, B., Nakamura, Y., DiGregorio, D.A., and Young, S.M., Jr. (2015). Ca^{2+} channel to synaptic vesicle distance accounts for the readily releasable pool kinetics at a functionally mature auditory synapse. J. Neurosci. *35*, 2083–2100.

Chicka, M.C., Hui, E., Liu, H., and Chapman, E.R. (2008). Synaptotagmin arrests the SNARE complex before triggering fast, efficient membrane fusion in response to Ca^{2+}. Nat. Struct. Mol. Biol. *15*, 827–835.

Christie, J.M., Chiu, D.N., and Jahr, C.E. (2011). Ca^{2+}-dependent enhancement of release by subthreshold somatic depolarization. Nat. Neurosci. *14*, 62–68.

Eggermann, E., and Jonas, P. (2011). How the 'slow' Ca^{2+} buffer parvalbumin affects transmitter release in nanodomain-coupling regimes. Nat. Neurosci. *15*, 20–22.

Eggermann, E., Bucurenciu, I., Goswami, S.P., and Jonas, P. (2011). Nanodomain coupling between Ca^{2+} channels and sensors of exocytosis at fast mammalian synapses. Nat. Rev. Neurosci. *13*, 7–21.

Ekerot, C.F., and Jörntell, H. (2003). Parallel fiber receptive fields: A key to understanding cerebellar operation and learning. Cerebellum *2*, 101–109.

Fox, M.A., and Sanes, J.R. (2007). Synaptotagmin I and II are present in distinct subsets of central synapses. J. Comp. Neurol. *503*, 280–296.

Geppert, M., Goda, Y., Hammer, R.E., Li, C., Rosahl, T.W., Stevens, C.F., and Südhof, T.C. (1994). Synaptotagmin I: A major Ca^{2+} sensor for transmitter release at a central synapse. Cell *79*, 717–727.

Giraudo, C.G., Eng, W.S., Melia, T.J., and Rothman, J.E. (2006). A clamping mechanism involved in SNARE-dependent exocytosis. Science *313*, 676–680.

Goswami, S.P., Bucurenciu, I., and Jonas, P. (2012). Miniature IPSCs in hippocampal granule cells are triggered by voltage-gated Ca^{2+} channels via microdomain coupling. J. Neurosci. *32*, 14294–14304.

Hefft, S., and Jonas, P. (2005). Asynchronous GABA release generates long-lasting inhibition at a hippocampal interneuron-principal neuron synapse. Nat. Neurosci. *8*, 1319–1328.

Herrmann, D.N., Horvath, R., Sowden, J.E., Gonzalez, M., Sanchez-Mejias, A., Guan, Z., Whittaker, R.G., Almodovar, J.L., Lane, M., Bansagi, B., et al. (2014). Synaptotagmin 2 mutations cause an autosomal-dominant form of Lambert-Eaton myasthenic syndrome and nonprogressive motor neuropathy. Am. J. Hum. Genet. *95*, 332–339.

Hosoi, N., Holt, M., and Sakaba, T. (2009). Calcium dependence of exo- and endocytotic coupling at a glutamatergic synapse. Neuron *63*, 216–229.

Hu, H., Gan, J., and Jonas, P. (2014). Interneurons. Fast-spiking, parvalbumin$^+$ GABAergic interneurons: From cellular design to microcircuit function. Science *345*, 1255263.

Hui, E., Bai, J., Wang, P., Sugimori, M., Llinas, R.R., and Chapman, E.R. (2005). Three distinct kinetic groupings of the synaptotagmin family: Candidate sensors for rapid and delayed exocytosis. Proc. Natl. Acad. Sci. USA *102*, 5210–5214.

Jackman, S.L., Turecek, J., Belinsky, J.E., and Regehr, W.G. (2016). The calcium sensor synaptotagmin 7 is required for synaptic facilitation. Nature *529*, 88–91.

Jarousse, N., and Kelly, R.B. (2001). The AP2 binding site of synaptotagmin 1 is not an internalization signal but a regulator of endocytosis. J. Cell Biol. *154*, 857–866.

Jones, M.V., Jonas, P., Sahara, Y., and Westbrook, G.L. (2001). Microscopic kinetics and energetics distinguish $GABA_A$ receptor agonists from antagonists. Biophys. J. *81*, 2660–2670.

Kerr, A.M., Reisinger, E., and Jonas, P. (2008). Differential dependence of phasic transmitter release on synaptotagmin 1 at GABAergic and glutamatergic hippocampal synapses. Proc. Natl. Acad. Sci. USA *105*, 15581–15586.

Kochubey, O., Babai, N., and Schneggenburger, R. (2016). A synaptotagmin isoform switch during the development of an identified CNS synapse. Neuron *90*, 984–999.

Koh, T.W., and Bellen, H.J. (2003). Synaptotagmin I, a Ca^{2+} sensor for neurotransmitter release. Trends Neurosci. *26*, 413–422.

Kraushaar, U., and Jonas, P. (2000). Efficacy and stability of quantal GABA release at a hippocampal interneuron-principal neuron synapse. J. Neurosci. *20*, 5594–5607.

Li, L., Bischofberger, J., and Jonas, P. (2007). Differential gating and recruitment of P/Q-, N-, and R-type Ca^{2+} channels in hippocampal mossy fiber boutons. J. Neurosci. *27*, 13420–13429.

Littleton, J.T., Stern, M., Perin, M., and Bellen, H.J. (1994). Calcium dependence of neurotransmitter release and rate of spontaneous vesicle fusions are altered in Drosophila synaptotagmin mutants. Proc. Natl. Acad. Sci. USA *91*, 10888–10892.

Liu, H., Dean, C., Arthur, C.P., Dong, M., and Chapman, E.R. (2009). Autapses and networks of hippocampal neurons exhibit distinct synaptic transmission phenotypes in the absence of synaptotagmin I. J. Neurosci. *29*, 7395–7403.

Liu, H., Bai, H., Xue, R., Takahashi, H., Edwardson, J.M., and Chapman, E.R. (2014). Linker mutations reveal the complexity of synaptotagmin 1 action during synaptic transmission. Nat. Neurosci. *17*, 670–677.

Mackler, J.M., Drummond, J.A., Loewen, C.A., Robinson, I.M., and Reist, N.E. (2002). The C_2B Ca^{2+}-binding motif of synaptotagmin is required for synaptic transmission *in vivo*. Nature *418*, 340–344.

Maximov, A., and Südhof, T.C. (2005). Autonomous function of synaptotagmin 1 in triggering synchronous release independent of asynchronous release. Neuron *48*, 547–554.

Mittelsteadt, T., Seifert, G., Alvárez-Barón, E., Steinhäuser, C., Becker, A.J., and Schoch, S. (2009). Differential mRNA expression patterns of the synaptotagmin gene family in the rodent brain. J. Comp. Neurol. *512*, 514–528.

Mittmann, W., Koch, U., and Häusser, M. (2005). Feed-forward inhibition shapes the spike output of cerebellar Purkinje cells. J. Physiol. *563*, 369–378.

Montesinos, M.S., Chen, Z., and Young, S.M., Jr. (2011). pUNISHER: A high-level expression cassette for use with recombinant viral vectors for rapid and long term in vivo neuronal expression in the CNS. J. Neurophysiol. *106*, 3230–3244.

Müller, C.S., Haupt, A., Bildl, W., Schindler, J., Knaus, H.G., Meissner, M., Rammner, B., Striessnig, J., Flockerzi, V., Fakler, B., and Schulte, U. (2010). Quantitative proteomics of the Cav2 channel nano-environments in the mammalian brain. Proc. Natl. Acad. Sci. USA *107*, 14950–14957.

Nagy, G., Kim, J.H., Pang, Z.P., Matti, U., Rettig, J., Südhof, T.C., and Sørensen, J.B. (2006). Different effects on fast exocytosis induced by synaptotagmin 1 and 2 isoforms and abundance but not by phosphorylation. J. Neurosci. *26*, 632–643.

Neher, E. (2015). Merits and limitations of vesicle pool models in view of heterogeneous populations of synaptic vesicles. Neuron *87*, 1131–1142.

Nishiki, T., and Augustine, G.J. (2004). Dual roles of the C2B domain of synaptotagmin I in synchronizing Ca^{2+}-dependent neurotransmitter release. J. Neurosci. *24*, 8542–8550.

Palmer, D., and Ng, P. (2003). Improved system for helper-dependent adenoviral vector production. Mol. Ther. *8*, 846–852.

Pang, Z.P., Melicoff, E., Padgett, D., Liu, Y., Teich, A.F., Dickey, B.F., Lin, W., Adachi, R., and Südhof, T.C. (2006a). Synaptotagmin-2 is essential for survival and contributes to Ca^{2+} triggering of neurotransmitter release in central and neuromuscular synapses. J. Neurosci. *26*, 13493–13504.

Pang, Z.P., Sun, J., Rizo, J., Maximov, A., and Südhof, T.C. (2006b). Genetic analysis of synaptotagmin 2 in spontaneous and Ca^{2+}-triggered neurotransmitter release. EMBO J. *25*, 2039–2050.

Pernía-Andrade, A.J., Goswami, S.P., Stickler, Y., Fröbe, U., Schlögl, A., and Jonas, P. (2012). A deconvolution-based method with high sensitivity and temporal resolution for detection of spontaneous synaptic currents in vitro and in vivo. Biophys. J. *103*, 1429–1439.

Poskanzer, K.E., Marek, K.W., Sweeney, S.T., and Davis, G.W. (2003). Synaptotagmin I is necessary for compensatory synaptic vesicle endocytosis in vivo. Nature *426*, 559–563.

Pouille, F., and Scanziani, M. (2001). Enforcement of temporal fidelity in pyramidal cells by somatic feed-forward inhibition. Science *293*, 1159–1163.

Roth, A., and Häusser, M. (2001). Compartmental models of rat cerebellar Purkinje cells based on simultaneous somatic and dendritic patch-clamp recordings. J. Physiol. *535*, 445–472.

Sakaba, T. (2008). Two Ca^{2+}-dependent steps controlling synaptic vesicle fusion and replenishment at the cerebellar basket cell terminal. Neuron *57*, 406–419.

Scheuss, V., Schneggenburger, R., and Neher, E. (2002). Separation of presynaptic and postsynaptic contributions to depression by covariance analysis of successive EPSCs at the calyx of Held synapse. J. Neurosci. *22*, 728–739.

Sommeijer, J.P., and Levelt, C.N. (2012). Synaptotagmin-2 is a reliable marker for parvalbumin positive inhibitory boutons in the mouse visual cortex. PLoS ONE *7*, e35323.

Südhof, T.C. (2002). Synaptotagmins: Why so many? J. Biol. Chem. *277*, 7629–7632.

Xu, J., Mashimo, T., and Südhof, T.C. (2007). Synaptotagmin-1, -2, and -9: Ca^{2+} sensors for fast release that specify distinct presynaptic properties in subsets of neurons. Neuron *54*, 567–581.

Young, S.M., Jr., and Neher, E. (2009). Synaptotagmin has an essential function in synaptic vesicle positioning for synchronous release in addition to its role as a calcium sensor. Neuron *63*, 482–496.

Zhang, J.Z., Davletov, B.A., Südhof, T.C., and Anderson, R.G. (1994). Synaptotagmin I is a high affinity receptor for clathrin AP-2: Implications for membrane recycling. Cell *78*, 751–760.

Cell Reports, Volume *18*

Supplemental Information

Synaptotagmin 2 Is the Fast Ca^{2+} Sensor at a Central Inhibitory Synapse

Chong Chen, Itaru Arai, Rachel Satterfield, Samuel M. Young, Jr., and Peter Jonas

Figure S1. Syt1 expression in cerebellar synapses of Syt2$^{+/+}$ and Syt2$^{-/-}$ mice, related to Figure 1.

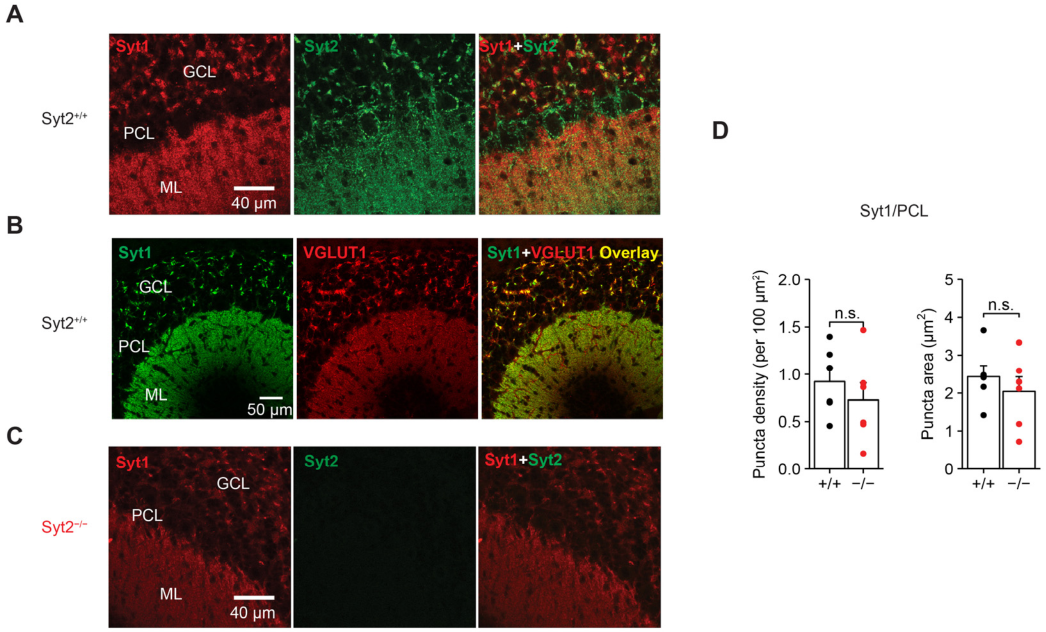

(A) Light-micrographs of cerebellar cortex, showing immunolabeling for Syt1 (left), Syt2 (center), and overlay (right).

(B) Light-micrographs of cerebellar cortex, showing immunolabeling for Syt1 (left), VGLUT1 (center), and overlay (right). Both (A) and (B) were obtained from Syt2$^{+/+}$ mice; single confocal sections.

(C) Similar analysis as in (A), but for Syt2$^{-/-}$ mouse.

(D) Summary bar graphs of puncta density (left) and puncta cross-sectional area (right) in the Purkinje cell layer (PCL). Bars represent mean ± SEM, circles indicate data from individual experiments. Note that both puncta density and area are very similar in Syt2$^{+/+}$ and Syt2$^{-/-}$ mice, suggesting lack of compensatory upregulation.

Figure S2. Similar infection and presynaptic protein levels for HdAd-Syt2 and HdAd-Syt1, related to Figure 5 and 6.

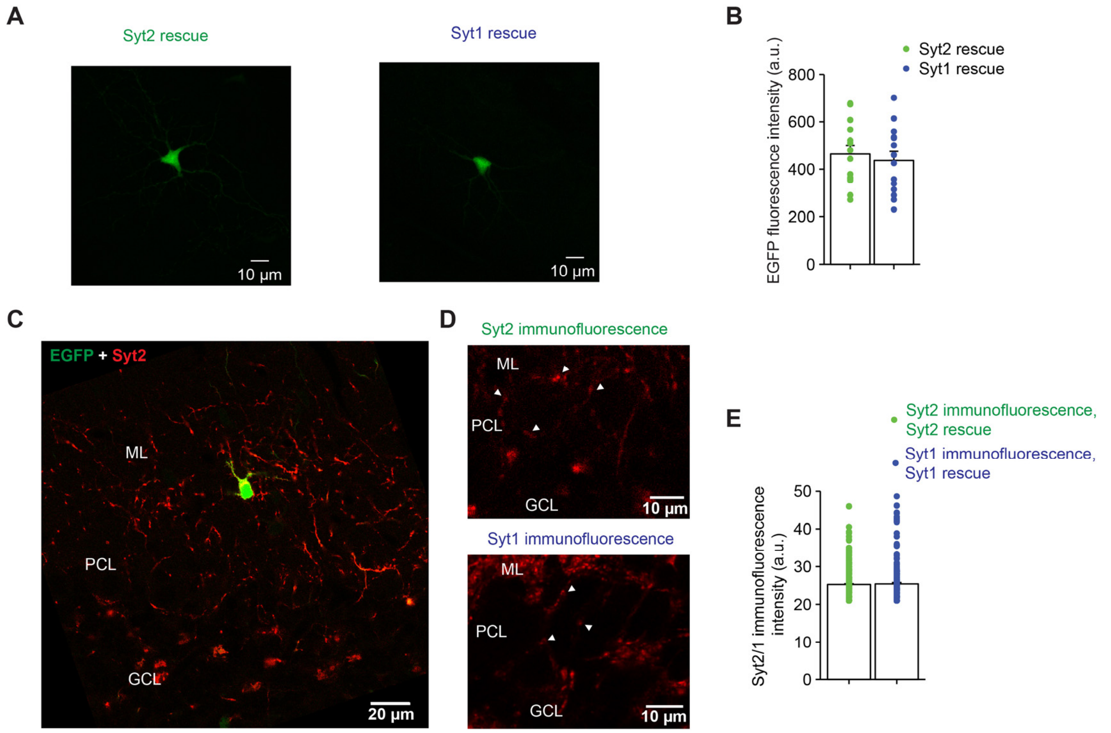

(A) Light-micrographs of cerebellar cortex, showing EGFP fluorescence in a BC in a Syt2$^{-/-}$ mouse after HdAd-Syt2 (left) or HdAd-Syt1 rescue (right).

(B) EGFP fluorescence intensity of cell bodies of HdAd-infected putative BCs in the inner molecular layer. Note that EGFP fluorescence intensity was similar (P = 0.63; 14 and 14 cells, respectively; confocal imaging performed with identical settings).

(C) Overview light micrograph of cerebellar cortex in a Syt2$^{-/-}$ mouse after infection with HdAd-Syt2. Green, EGFP fluorescence; red, Syt2 immunoreactivity. Single confocal section.

(D) High resolution light micrographs of Syt2 immunoreactivity after Syt2 rescue (top) and Syt1 immunoreactivity after Syt1 rescue (bottom) in the Purkinje cell layer. Note that both synaptotagmins are expressed after viral rescue, whereas synaptotagmin immunoreactivity is absent in Syt2$^{-/-}$ animals without rescue (Figure S1C).

(E) Quantitative analysis of Syt2 immunofluorescence after Syt2 rescue (green) and Syt1 immunofluorescence after Syt1 rescue (blue) in Syt2$^{-/-}$ animals. Measurements were made in presynaptic terminals on Purkinje cell somata, representing putative BC terminals. Fluorescence intensity was determined in the same slices following double injections into the same animal under similar conditions (identical laser illumination,

identical detector gain, similar distance from slice surface). Note that the fluorescence intensities were similar for Syt2 and Syt1 ($P = 0.58$).

Figure S3. Rescue of functional properties of synaptic transmission with HdAd-Syt2 and HdAd-Syt1, related to Figure 5 and 6.

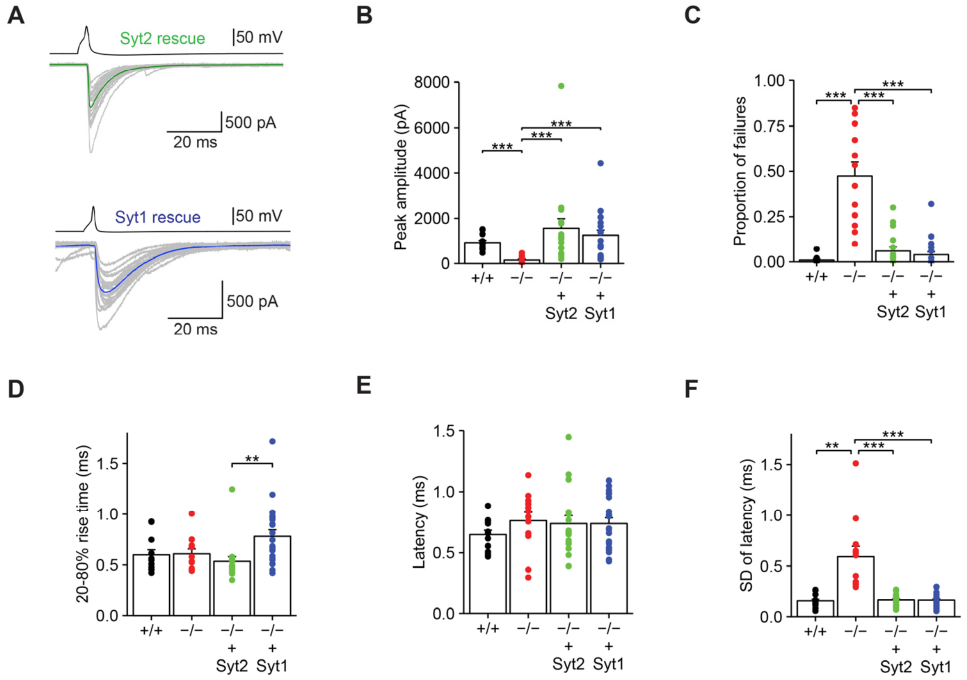

(A) Original traces of unitary IPSCs after infection of the presynaptic neuron with HdAd-Syt2 (left) or HdAd-Syt1 (right). Upper traces represent presynaptic AP, lower traces show 20 consecutive IPSC traces (gray), superimposed with the average (green and blue, respectively). Note that HdAd-Syt2 and HdAd-Syt1 were equally effective in rescuing the peak amplitude of evoked IPSCs.

(B–F) Comparison of IPSC peak amplitude (B), proportion of failures (C), 20–80% rise time (D), latency (E), and standard deviation of latency (F). Black, data from Syt2$^{+/+}$ synapses; red, data from Syt2$^{-/-}$ synapses; green, HdAd-Syt2 rescue; blue, HdAd-Syt1 rescue (both on Syt2$^{-/-}$ background). Bars represent mean ± SEM, circles indicate data from individual experiments. Note that both HdAd-Syt2 and HdAd-Syt1 similarly rescued peak amplitude, proportion of failures, latency, and standard deviation of latency, but differentially rescued the 20–80 % rise time.

Figure S4. Determining quantal size at BC–PC synapses, related to Figure 6.

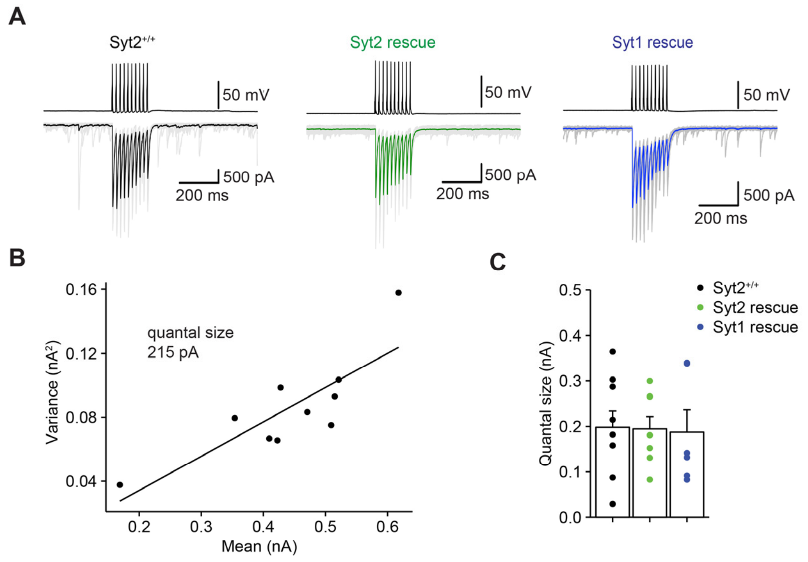

(A) Evoked IPSCs following a 50-Hz train of ten stimuli given to the presynaptic BC. Left, Syt2$^{+/+}$; center, Syt2 rescue; right, Syt1 rescue (both in Syt2$^{-/-}$ mice).

(B) Plot of variance against mean amplitude for each IPSC evoked by train stimulation. Data from a representative experiment (from a Syt2$^{+/+}$ mouse). Data points were fit by linear regression. The line slope, corresponding to quantal size, was 215 pA. Linearity of the data is consistent with constant quantal size during train stimulation.

(C) Summary bar graph of quantal size for Syt2$^{+/+}$, HdAd-Syt2 rescue, and HdAd-Syt1 rescue (both in Syt2$^{-/-}$ mice). Data from 9, 8, and 6 pairs.

Figure S5. Estimates of pool size and refilling rates are unlikely to be distorted by postsynaptic receptor saturation or desensitization, related to Figure 6.

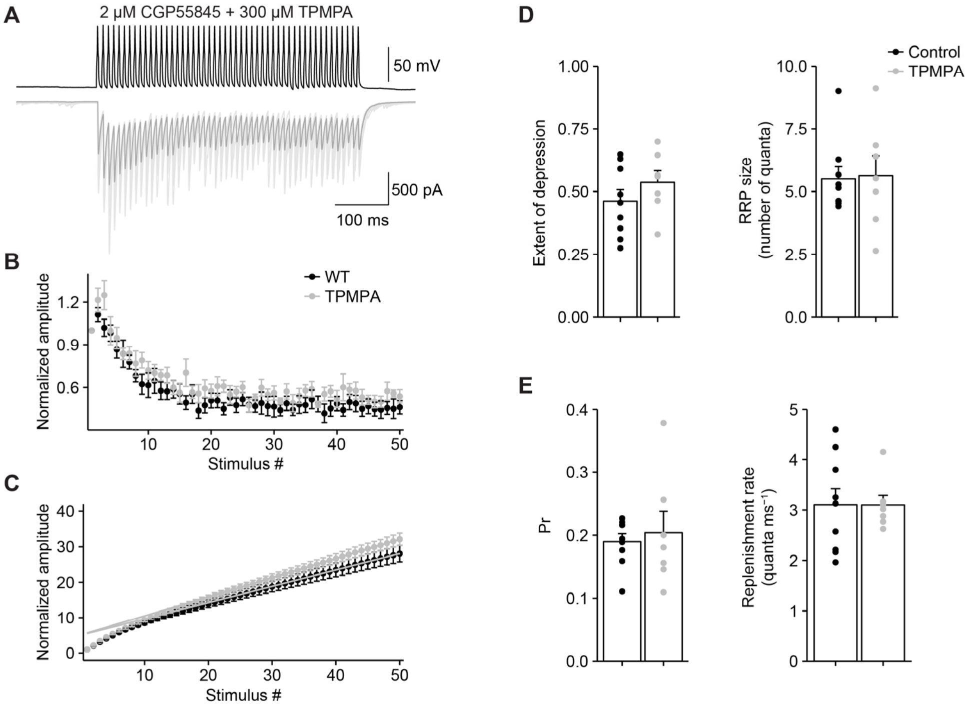

(A) Presynaptic APs (top) and evoked IPSCs (bottom) in the presence of 300 μM of low-affinity $GABA_A$ receptor antagonist TPMPA. 2 μM CGP55845 was further added to minimize effects of TPMPA on transmitter release via presynaptic $GABA_B$ receptors. 10 consecutive individual traces (light gray) and the corresponding average trace (dark gray) are shown superimposed.

(B, C) Plot of normalized IPSC peak amplitude against stimulus number (B, non-cumulative amplitude; C, cumulative plot). Black, data in control; gray, in the presence of 300 μM TPMPA. Lines represent the results of linear regression of last ten data points. Note that the pool size, as indicated by the intersection of the regression line with the ordinate, is almost identical in the absence and presence of TPMPA.

(D, E) Summary bar graphs of the extent of depression after 50 APs (D, left), size of the readily releasable pool (RRP; D, right), release probability (P_r; E, left), and replenishment rate (E, right). Bars indicate mean ± SEM; solid circles represent data from individual experiments. Data from 9 and 7 pairs, respectively. Note that the estimates were similar for control and TPMPA (P = 0.3, 0.76, 0.76, and > 0.99). All data were obtained from wild-type synapses.

Figure S6. Reversal potential of monosynaptic EPSCs and disynaptic IPSCs evoked by parallel fiber stimulation, related to Figure 7.

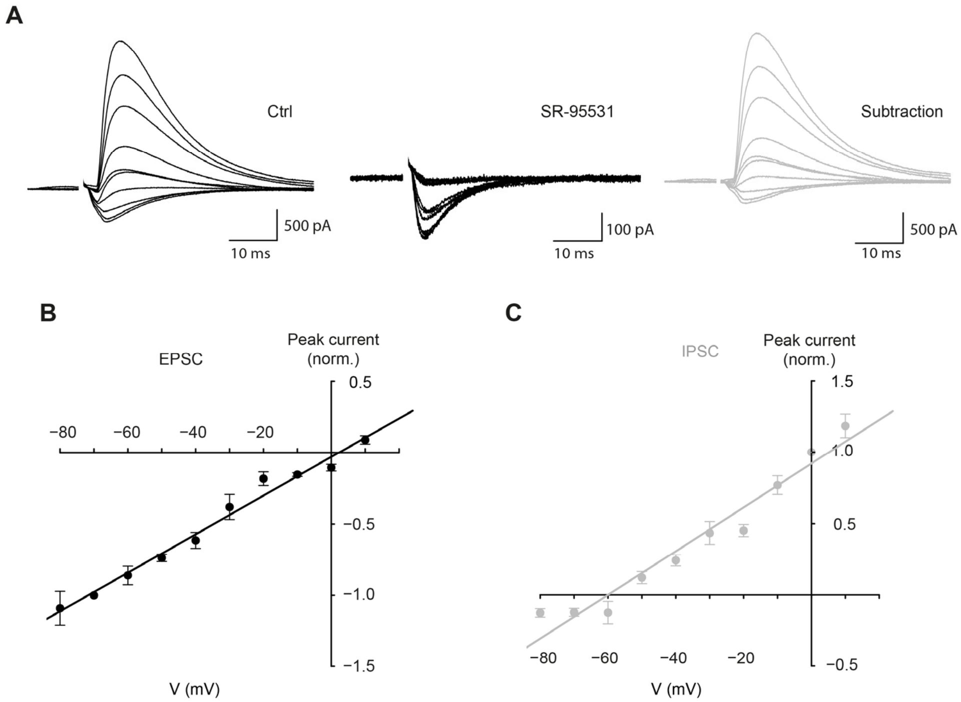

(A) Left, recording of mixed EPSCs and IPSCs; center, recording of EPSCs in the presence of 10 µM SR-95531; right, traces obtained by digital subtraction; traces were recorded at membrane potentials from −80 mV to +10 mV in 10-mV steps in a PC.

(B) Current-voltage relation for pharmacologically isolated EPSCs.

(C) Current-voltage relation for IPSCs isolated by digital subtraction. Data points indicate mean values from 4 experiments. Current amplitudes were normalized to the values at −70 mV and 0 mV, respectively. Data points were fit by linear regression, giving reversal potentials of 3.1 mV (B) and −60.0 mV (C).

Figure S7. Mechanisms of fast signaling at cerebellar BC–PC synapses, related to Figure 1–7.

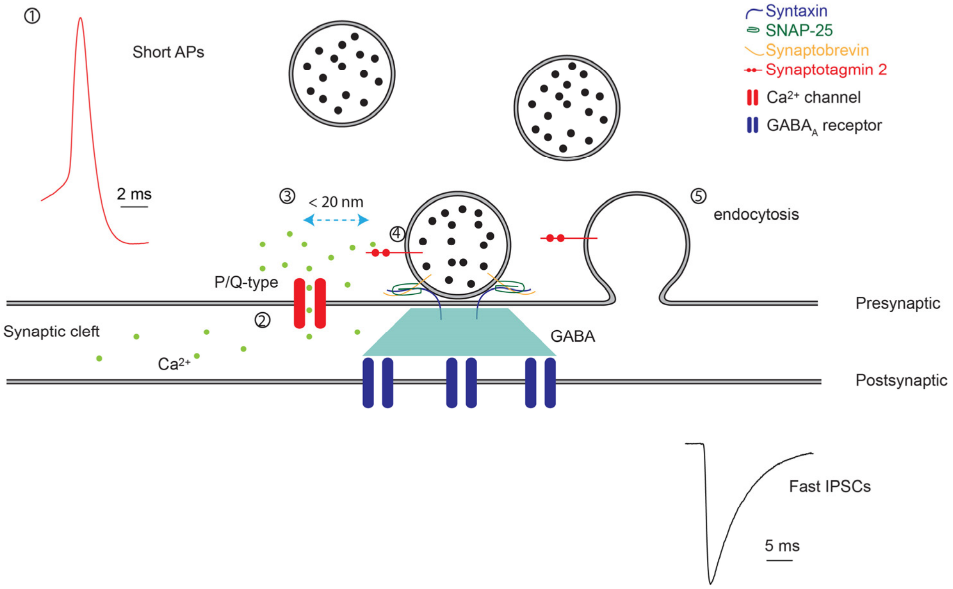

Upper left, AP recorded in a presynaptic BC. Lower right, IPSCs recorded in a postsynaptic PC. Mechanisms of rapid signaling include: (1) Short duration of presynaptic AP, (2) fast gating of presynaptic P/Q-type Ca^{2+} channels (Arai and Jonas, 2014; Li et al., 2007), (3) short coupling distance between presynaptic Ca^{2+} channels and release sensor (synaptotagmin 2, red) (Arai and Jonas, 2014), (4) fast kinetics of Syt2-mediated exocytosis (present paper), and (5) fast replenishment of releasable vesicle pool due to Syt2-mediated endocytosis (present paper).

Table S1. Functional properties of synaptic transmission at cerebellar BC–PC synapses in $Syt2^{+/+}$ and $Syt2^{-/-}$ mice, related to Figure 2, 3, and 4.

**Parameter**	**$Syt2^{+/+}$**	**$Syt2^{-/-}$**	**P**
Latency	0.65± 0.04 ms (12)	0.75 ± 0.07 ms (12)	0.1135
SD Latency	0.15± 0.02 ms (12)	0.59 ± 0.10 ms (12)	< 0.0001
20–80% rise time	0.60 ± 0.05 ms (12)	0.64 ± 0.05 ms (12)	0.5137
IPSC peak amplitude	925.1 ± 99.2 pA (12)	151.9 ± 44.2 pA (12)	< 0.0001
IPSC decay time constant	9.19 ± 0.51 ms (12)	9.53 ± 1.08 ms (12)	0.6707
Proportion of failures	0.91 ± 0.58% (12)	47.4 ± 7.7% (12)	< 0.0001
Paired-pulse ratio $IPSC_2$ / $IPSC_1$	1.06 ± 0.05 (11)	1.79 ± 0.16 (16)	0.0003
$IPSC_{10}$ / $IPSC_1$	0.61 ± 0.02 (11)	2.79 ± 0.46 (16)	< 0.0001
Miniature IPSC frequency	3.75 ± 0.42 Hz (12)	9.64 ± 1.74 Hz (13)	0.0008
Miniature IPSC peak amplitude	110.0 ±11.0 pA (12)	141.7±12.0 pA (13)	0.0868

Mean ± SEM (n, number of experiments).

Table S2. Differential helper-dependent adenovirus-mediated rescue by Syt2 and Syt1 in $Syt2^{-/-}$ mice, related to Figure 5 and 6.

Parameter	HdAd-Syt2	HdAd-Syt1	P
Latency	0.74 ± 0.06 ms (17)	0.74 ± 0.05 ms (21)	>0.9999
SD latency	0.16 ± 0.01 ms (17)	0.16 ± 0.01 ms (21)	>0.9999
20–80% rise time	0.534 ± 0.05 ms (17)	0.781 ± 0.065 ms (21)	0.0026
IPSC peak amplitude	1559.3 ± 426.4 pA (17)	1250.3 ± 225.2 pA (21)	0.9740
IPSC decay time constant	7.71 ± 0.41 ms (17)	9.27 ± 0.55 ms (21)	0.2700
Proportion of failures	5.91 ± 2.28% (17)	3.81 ± 1.67% (21)	>0.9999
Paired-pulse ratio $IPSC_2$ / $IPSC_1$	1.01 ± 0.04 (10)	0.82 ± 0.05 (10)	0.0294
$IPSC_{10}$ / $IPSC_1$	0.67 ± 0.08 (10)	0.39 ± 0.032 (10)	0.0030
Recovery from depression τ	2.17 ± 0.31 s (13)	4.89 ± 0.79 s (12)	0.011

All experiments were performed in $Syt2^{-/-}$ mice.
Mean ± SEM (n, number of experiments).

Data included in this paper are based on recordings from a total of 137 BC–PC paired recordings (32 for experiments on $Syt2^{+/+}$, 28 for $Syt2^{-/-}$, 37 for HdAd-Syt2 rescue, and 40 for HdAd-Syt1 rescue) and 48 PC recordings (25 for mIPSC and 23 for disynaptic inhibition experiments).
